# The Australian Traumatic Brain Injury Initiative: Systematic Review of Clinical Factors Associated with Outcomes in People with Moderate-Severe Traumatic Brain Injury

**DOI:** 10.1089/neur.2023.0111

**Published:** 2024-07-04

**Authors:** Ancelin McKimmie, Jemma Keeves, Adelle Gadowski, Matthew K. Bagg, Ana Antonic-Baker, Amelia J. Hicks, Regina Hill, Nyssa Clarke, Andrew Holland, Bill Veitch, Daniel Fatovich, Sandy Reeder, Lorena Romero, Jennie L. Ponsford, Natasha A. Lannin, Terence J. O’Brien, D. Jamie Cooper, Nick Rushworth, Melinda Fitzgerald, Belinda J. Gabbe, Peter A. Cameron, Australian Traumatic Brain Injury Initiative Investigators, Tara Alexander, Vicki Anderson, Ana Antonic-Baker, Elizabeth Armstrong, Franz E Babl, Matthew K Bagg, Zsolt J Balogh, Karen M Barlow, Judith Bellapart, Niranjan Bidargaddi, Erika Bosio, Peter Bragge, Michael Bynevelt, Karen Caeyenberghs, Peter A Cameron, Jacquelin Capell, Kevin E K Chai, Lyndsey E Collins-Praino, D J Jamie Cooper, Gill Cowen, Louise M Crowe, Tim Cudmore, Jennifer Cullen, Kate Curtis, Anthony Delaney, Graeme Dibdin, Sandra Eades, Gary F Egan, Daniel Y Ellis, Ari Ercole, Daniel M Fatovich, Murray J Fisher, Mark Fitzgerald, Melinda Fitzgerald, Jennifer Fleming, Roslyn Francis, Belinda J Gabbe, Adelle Gadowski, John Gilroy, Mitchell A Hansen, James E Harrison, Luke J Haseler, Leanne Hassett, Sarah C Hellewell, Amelia J Hicks, Andrew F Hill, Andrew J A Holland, Stephen Honeybul, Rosalind L Jeffree, Chris Joyce, Elizabeth Kendall, Kate King, Natasha A Lannin, Meng Law, Andrew I R Maas, Adam Mahoney, Peter Makin, Peter Mayhew, Alison McDonald, Skye McDonald, Stuart J McDonald, Ancelin McKimmie, Robert McNamara, Shiv Meka, David K Menon, Gary Mitchell, Rowena Mobbs, Fatima A Nasrallah, Virginia F J Newcombe, Terence J O’Brien, John H Olver, Gerard M O’Reilly, Tamara Ownsworth, Paul M Parizel, Michael Parr, Jennie L Ponsford, Bruce Powell, Patricia Ratajczak, Michael C Reade, Sandy Reeder, Christopher Reid, Julia Robertson, Suzanne Robinson, Danette Rowse, Stephen E Rose, Jeffrey V Rosenfeld, Jason P Ross, Nick Rushworth, Adam Scheinberg, Bridgette D Semple, Sandy R Shultz, Grahame K Simpson, Warwick J Teague, Leanne Togher, Andrew A Udy, Kirsten Vallmuur, Dinesh Varma, James Vickers, Janet Wagland, James Walsham, Adam J Wells, Luke Whiley, Gavin Williams, Jodie K Williams, Roslind Witham, David K Wright, Louise York, Jesse T Young, Heidi Zeeman

**Affiliations:** ^1^ School of Public Health and Preventive Medicine, Monash University, Melbourne, Australia.; ^2^ Faculty of Health Sciences, Curtin Health Innovation Research Institute, Curtin University, Bentley, Australia.; ^3^ Perron Institute for Neurological and Translational Science, Nedlands, Australia.; ^4^ Centre for Pain IMPACT, Neuroscience Research Australia, Sydney, Australia.; ^5^ School of Health Sciences, University of Notre Dame Australia, Fremantle, Australia.; ^6^ Department of Neuroscience, Central Clinical School, Monash University, Melbourne, Australia.; ^7^ Monash-Epworth Rehabilitation Research Centre, Epworth Healthcare, Melbourne, Australia.; ^8^ School of Psychological Sciences, Monash University, Melbourne, Australia.; ^9^ Regina Hill Effective Consulting Pty Ltd, Melbourne, Australia.; ^10^ Faculty of Medicine and Health, The Children’s Hospital at Westmead Clinical School, University of Sydney School of Medicine, Westmead, Australia.; ^11^ Emergency Medicine, Royal Perth Hospital, University of Western Australia, Perth, Australia.; ^12^ Centre for Clinical Research in Emergency Medicine, Harry Perkins Institute of Medical Research, Nedlands, Australia.; ^13^ Alfred Health, Melbourne, Australia.; ^14^ School of Public Health and Preventive Medicine, Australian and New Zealand Intensive Care Research Centre, Monash University, Melbourne, Australia.; ^15^ Department of Intensive Care and Hyperbaric Medicine, Melbourne, Australia.; ^16^ Brain Injury Australia, Sydney, Australia.; ^17^ Health Data Research UK, Swansea University Medical School, Swansea University, Singleton Park, United Kingdom.; ^18^ National Trauma Research Institute, Melbourne, Australia.; ^19^ Emergency and Trauma Centre, The Alfred Hospital, Melbourne, Australia.

**Keywords:** common data elements, critical care, emergency medical services, Glasgow Coma Scale, health care outcome assessment, MeSH term, multiple trauma, physical examination, systematic review, trauma severity indices, traumatic brain injuries, vital signs

## Abstract

The aim of the Australian Traumatic Brain Injury Initiative (AUS-TBI) is to design a data dictionary to inform data collection and facilitate prediction of outcomes for moderate-severe traumatic brain injury (TBI) across Australia. The process has engaged diverse stakeholders across six areas: social, health, clinical, biological, acute interventions, and long-term outcomes. Here, we report the results of the clinical review. Standardized searches were implemented across databases to April 2022. English-language reports of studies evaluating an association between a clinical factor and any clinical outcome in at least 100 patients with moderate-severe TBI were included. Abstracts, and full-text records, were independently screened by at least two reviewers in Covidence. The findings were assessed through a consensus process to determine inclusion in the AUS-TBI data resource. The searches retrieved 22,441 records, of which 1137 were screened at full text and 313 papers were included. The clinical outcomes identified were predominantly measures of survival and disability. The clinical predictors most frequently associated with these outcomes were the Glasgow Coma Scale, pupil reactivity, and blood pressure measures. Following discussion with an expert consensus group, 15 were recommended for inclusion in the data dictionary. This review identified numerous studies evaluating associations between clinical factors and outcomes in patients with moderate-severe TBI. A small number of factors were reported consistently, however, how and when these factors were assessed varied. The findings of this review and the subsequent consensus process have informed the development of an evidence-informed data dictionary for moderate-severe TBI in Australia.

**Figure:**
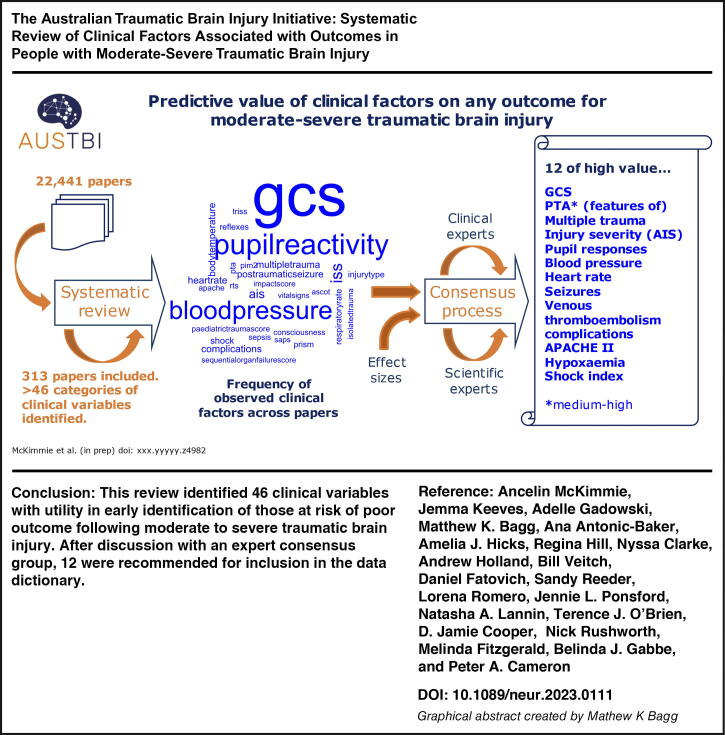

## Introduction

Traumatic brain injury (TBI) is a major cause of disability and death in Australia and globally.^1,2^ TBI can involve significant health care resources and may also result in dramatic and long-lasting consequences for patients, their families, and care givers.^1,3^

TBI is not a single entity, and the cause, pathology, severity, and prognosis of presentations varies greatly.^4^ The rate and degree of recovery following moderate-severe TBI is variable, and predicting outcomes following TBI remains imprecise.^5^ Clinical decision making is inconsistent and survival and functional outcomes following TBI are not improving despite decades of research in this area.^5,6^

Developing the evidence base for the association between a range of acute care clinical factors and outcomes may help in developing predictive modeling, which improves care pathways and outcomes for patients with moderate-severe TBI.^1^ Systematic reviews addressing a broad range of acute clinical factors can provide insight as to potentially predictive indicators for inclusion in predictive modeling.

The clinical factors included in the scope of this systematic review were clinical observations, mechanism of injury, injury severity, diagnoses, and severe complications and were focused in the acute care setting in order to identify potentially predictive indicators that could be incorporated in clinical tools for acute care.

The Australian Traumatic Brain Injury Initiative (AUS-TBI) seeks to improve prediction models, health care, and outcomes for people with moderate-severe TBI in Australia.^7^ A key objective of AUS-TBI was the development of a *data dictionary* (a set of common data elements—units of data with well-defined attributes—that constitute the ontology for a coherent data structure) to facilitate data collection and enable improved prediction of outcomes of people who experience moderate-severe TBI in Australia.^8^ This review was one of a series of articles describing the national approach used to select the common data elements that have been used to predict outcome following TBI across the lifespan. This series describes the development of the data dictionary including the consensus processes across six study domains: (1) demographic, injury event, and social characteristics, (2) pre-existing health conditions, (3) the clinical experience, (4) biological mechanisms, (5) acute interventions, and (6) longer-term outcomes. This article reports the findings for the clinical review domain only, while accompanying articles in this series report the activities in the other five study areas.

## Methods

This systematic review was prospectively registered on PROSPERO (CRD42022297902). The study is reported herein with respect to the 2020 PRISMA statement.^9^

### Objectives

The objectives of this study were to:

1. Identify published records of studies evaluating clinical factors associated with outcomes in people with moderate-severe TBI.
2. Identify unique clinical factors evaluated by studies in the record set.
3. Assign judgments of predictive value to each observed association between clinical factors and clinical outcomes.

### Outcomes

The primary outcome of this study was the set of clinical factors predictive of outcome. The secondary outcomes were the set of clinical outcomes and the set of unique studies.

### Sampling

Standardized, piloted, search strategies were used to search eight bibliographic databases (Central, Cinahl, Embase, Emcare, Medline, Scopus, SportDiscus, and Web of Science) from inception to April 2022. Full details of the methods followed for this review are described in Gabbe et al. (series article 2) unless otherwise stated.^10^ To summarize, two independent team members screened the title and abstract of each record, resolving any disagreements through discussion with a third independent team member if required. Full-length records were screened in duplicate to confirm inclusion for data extraction.

Specific to this systematic review, only studies with a sample size of at least 100 patients with moderate-severe TBI were included. Clinical factors were operationalized biomedical parameters hypothesized to influence or predict health outcomes. The clinical factors in the scope of this review included clinical observations (e.g., heart rate, blood pressure), injury event details (e.g., severity, injury type), diagnoses, and severe complications (e.g., acute lung injury, hospital acquired pneumonia), measured at the time of injury or during acute care. Clinical outcomes were operationalized broadly as any dependent variable reflective of the clinical features or lived experience of TBI, and included measurable changes in function, quality of life, and survival outcomes. Moderate-severe TBI was defined as the reported presence of at least one of, medically confirmed, (1) initial or lowest Glasgow Coma Scale (GCS) <13, (2) post-traumatic amnesia (PTA) duration >24 h, or (3) abnormal findings on head computed tomography (CT). This operational definition included the complicated “mild” injury type (GCS 13–15), with intracranial findings on neuroimaging.

Records of studies that included patients without TBI, or with mild, non-complicated TBI were excluded unless the data on participants with moderate-severe TBI were reported separately from other participants or if ≥80% of the sample had moderate-severe TBI. There were no restrictions based on patient demographic characteristics. The settings in which the study occurred were restricted to acute care, which included pre-hospital and acute inpatient settings; sub-acute care and rehabilitation settings were excluded.

### Data extraction

The co-ordinating team members and AUS-TBI Steering Committee co-designed the table of items (variables) for data extraction from included study reports. Standardized data sheets were built in Google Sheets (GSuite, Monash University), piloted, and then adapted to the requirements of this review. Detailed data extraction methods are described in Gabbe et al. (series article 2).^10^ Briefly, the data items captured information on study characteristics, measured predictors variables (clinical factors), measured clinical outcomes, baseline sample size, subgroups, covariate adjustment, and reported measures of effects. Study authors were not contacted to request missing or clarify uncertain data for this iteration of the review.

Data were extracted from each record by a single team member. Expert judgment was used to identify predictor:outcome associations, i.e., associations between a clinical factor and a clinical outcome evaluated in each study. The sheets included additional data items that summarized or structured the information in the extracted data items. These were filled during the extraction process. Upon completion, the team member used a pre-defined decision algorithm to assign a judgment of predictive value to each observed predictor:outcome association. Table 1 outlines the decision algorithm for judgments and defines high, medium, and low predictive values. This review did not assess study quality as the purpose was to identify possible clinical factors that had been reported as useful in predicting outcomes, not to assess methodological quality of the particular studies. Data from a random 25% of records were extracted in duplicate and compared for consistency. Disagreements were resolved in discussion, or by a third team member.

**Table 1.** Decision algorithm for judgments of predictive value

HIGH: large sample size AND association tested in whole study sample AND adjusting for covariates AND a strong predictive relationship was observed.
MEDIUM: association tested in whole sample AND limited adjustment for covariates OR moderate predictive relationship.
LOW: association tested in subset of sample OR limited predictive relationship observed.

**Table 2.** Associations Between Clinical Factors and Clinical Outcomes

Clinical factor studied^^^	Outcomes assessed against	Number of studies(*n* high value/*n* all studies)	Types of study design
AIS^16^	Cognitive outcome, complications, CT (contusion), discharge destination, employment, functional outcome, GCS, GOSE, HPA function, intubation, mortality, post-traumatic stroke	1/22	Prospective cohort, retrospective cohort
APACHE^a^	Functional status scale, GOSE, mortality	0/3	Prospective cohort, retrospective cohort
ASCOT^b^	Discharge destination	0/1	Prospective cohort
Blood pressure^17–20^	Admission to ICU, complications, consciousness, FIM, GCS, GOSE, LOS, mortality, PCPCS, post-traumatic seizure, post-traumatic stroke, quality of life, transport, trauma center care	4/87	Retrospective cohort, prospective cohort, retrospective cross-sectional
Body temperature^21^	Complications, FIM, GCS, GOSE, mortality	1/13	Retrospective cohort
Complications^c^	Complications, GOSE, mortality, post-traumatic stroke	0/12	Retrospective cohort, retrospective case-control, cross-sectional case control, prospective cohort
GCS^d^	Mortality, Disability Rating Scale (DRS), complications, GOSE, CT (mass effect), functional outcome, discharge destination, post-traumatic seizure, employment, CT (contusion), intubation, neurosurgery, consciousness, coagulopathy, post-traumatic stroke, refractory intracranial hypertension, Functional Status Scale, FIM, ICU admission, LOS, PCPCS, trauma center care, CT (EDH), CT (ICH), CT (SDH), surgery, Quality of Life (QoL), transport, cervical spine injury	0/227	Retrospective cohort, randomized, controlled trial, retrospective case-control, prospective cohort
Heart rate^e^	CT (lesion), mortality, discharge destination, GOSE, post-traumatic seizure	0/9	Retrospective cohort, prospective cohort
IMPACT Prognostic Score^f^	GOSE, GCS	0/1	Prospective cohort
Injury type^g^	Mortality, functional outcome, GOSE, LOS	0/8	Retrospective cohort, prospective cohort
Isolated trauma^h^	Mortality, LOS	0/2	Retrospective cohort
Injury severity score^22–25^	Mortality, GOSE, CT (lesion), complications, CT (mass effect), HPA function, cervical spine injury, coagulopathy, functional outcome, discharge destination, post-traumatic seizure, neurosurgery	4/50	Retrospective cohort, prospective cohort
Multiple trauma^i^	Mortality, functional outcome, GOSE	0/15	Retrospective cohort, prospective cohort
PIM 2^j^	GOSE	0/2	Retrospective cohort
Pediatric Trauma Score^k^	Mortality, PCPCS	0/3	Retrospective cohort
Post-traumatic seizure^l^	Mortality, GOSE	0/3	Retrospective cohort
PRISM^m^	GOSE	0/3	Retrospective cohort
PTA^n^	Mortality, GOSE, neurosurgery, PCPCS	0/5	Retrospective cohort, prospective cohort, prospective case control
Pupil reactivity^24–33^	Mortality, GOSE, functional outcome, trauma center care, transport, post-traumatic stroke, neurosurgery, complications, PCPCS, consciousness, LOS, FIM, GCS	10/101	Retrospective cohort, retrospective case control, prospective cohort
Reflexes^o^	Mortality, post-traumatic stroke, GOSE, discharge destination	0/5	Retrospective cohort, prospective cohort
Respiratory rate^p^	Mortality, post-traumatic stroke	0/4	Retrospective cohort, prospective cohort
RTS^q^	Mortality, GOSE	0/6	Retrospective cohort, prospective cohort
SAPS^r^	GOSE	0/1	Retrospective cohort
Sepsis^s^	Mortality	0/2	Retrospective cohort, prospective cohort
SOFS^t^	Mortality	0/1	Retrospective cohort
Shock^u^	Mortality, complications, intubation, LOS, GCS	0/10	Retrospective cohort, prospective cohort
TRISS^v^	Mortality, transport	0/3	Retrospective cohort, prospective cohort
Vital signs^22^	LOS	1/1	Retrospective cohort

^^^References only included for studies deemed as high predictive value. For other clinical factors, an example article has been referenced in the footnote, including the highest level of predictive value for that clinical factor and any outcome.

^a^ APACHE^34^ (medium).

^b^ ASCOT^35^ (low).

^c^ Complications^36^ (medium).

^d^ GCS^37^ (medium).

^e^ Heart rate^38^ (low).

^f^ IMPACT score^39^ (null).

^g^ Injury type^40^ (medium).

^h^ Isolated trauma^41^ (null).

^i^ Multiple trauma^42^ (medium).

^j^ PIM 2^43^ (medium).

^k^ Pediatric Trauma Score^44^ (low).

^l^ Post-traumatic seizure^45^ (medium).

^m^ PRISM^46^ (low).

^n^ PTA^47^ (low).

^o^ Reflexes^48^ (low).

^p^ Respiratory rate^49^ (low).

^q^ RTS^43^ (medium).

^r^ SAPS^50^ (low).

^s^ Sepsis^42^ (low).

^t^ SOFS^51^ (low).

^u^ Shock^52^ (medium).

^v^ TRISS^53^ (low).

AIS, Abbreviated Injury Scale; GCS, Glasgow Coma Scale; GOSE, Glasgow Outcome Scale-Extended; HPA, hypothalamic pituitary adrenal axis; APACHE, Acute Physiology and Chronic Health Evaluation; ASCOT, A Severity Characterisation of Trauma; ICU, intensive care unit; FIM, Functional Independence Measure; LOS, length of stay; PCPCS, Paediatric Cerebral Performance Category Scale; EDH, epidural hematoma; ICH, intracerebral hemorrhage; SDH, subdural hematoma; PIM 2, Paediatric Index of Mortality 2; PRISM, Paediatric Risk of Mortality Score; PTA, post-traumatic amnesia; RTS, Revised Trauma Score; SAPS, Simplified Acute Physiology Score; SOFS, Sequential Organ Failure Score; TRISS, Trauma Score and Injury Severity Score.

### Data management

Completed datasheets were locked to editing, mirrored to static versions on OneDrive (Microsoft 365), and read to R (version 4.3.1). Data were inspected, cleaned, and then summarized using the tidyverse.^11^ The extent of missing values was calculated for each variable. Categorical variables were summarized using the frequency of observations on each level. Variables that captured semistructured text were coerced to factors (the categorical structure in R) and unique levels identified through homogenization by the review team. Original free text was preserved. The subsequently “cleaned” dataset was written out to .csv, with values locked to editing.

Unique clinical factors and outcomes were displayed, with respect to their observed frequency across included studies, in word clouds (Figs. 2 and 3). The associations between clinical factors and clincial outcomes are presented in Table 2.

**FIG. 1.**
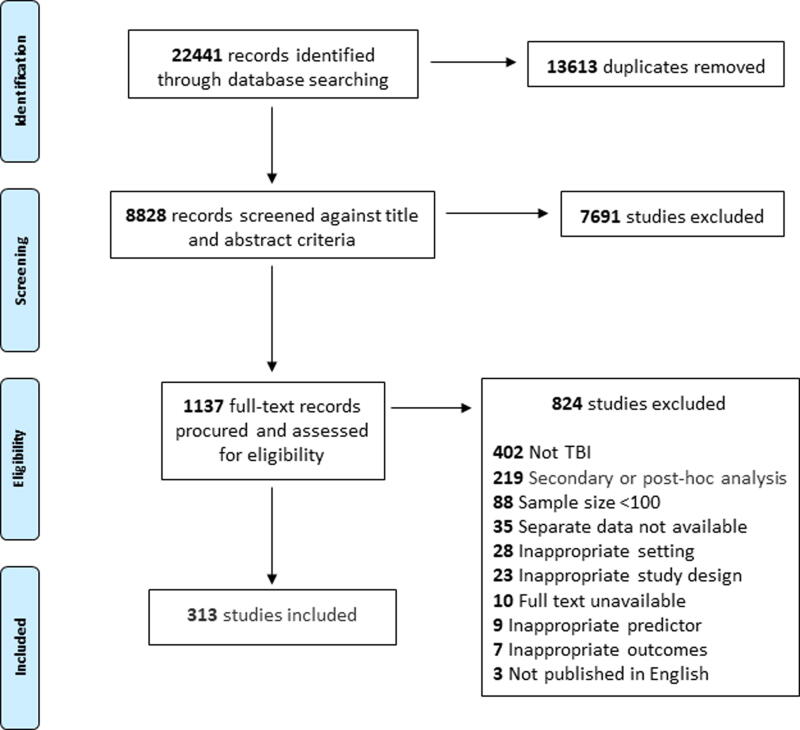
PRISMA flow diagram.

**FIG. 2.**
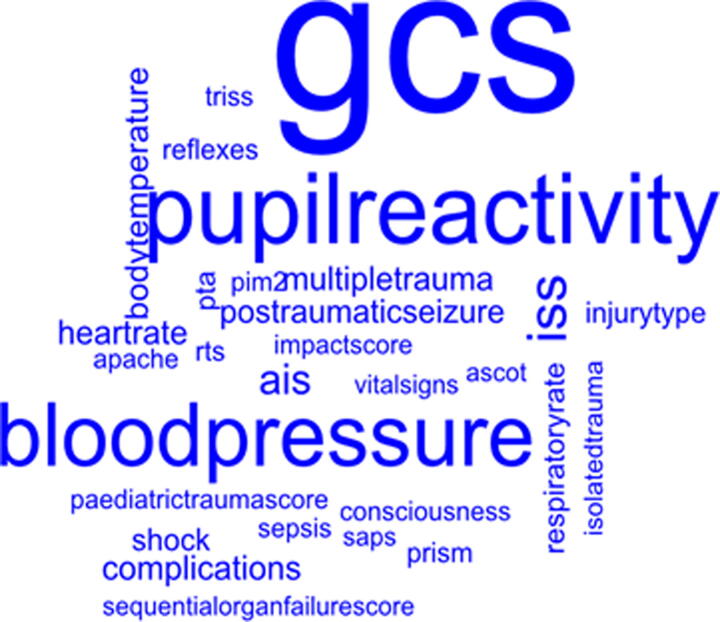
Word cloud of clinical predictors of outcome (size of word denotes frequency in included records). AIS, Abbreviated Injury Scale; ASCOT, A Severity Characterisation of Trauma; APACHE, Acute Physiology and Chronic Health Evaluation; PRISM, Paediatric Risk of Mortality Score; PTA, post-traumatic amnesia; RTS, Revised Trauma Score; SAPS, Simplified Acute Physiology Score (SAPS) II.

**FIG. 3.**
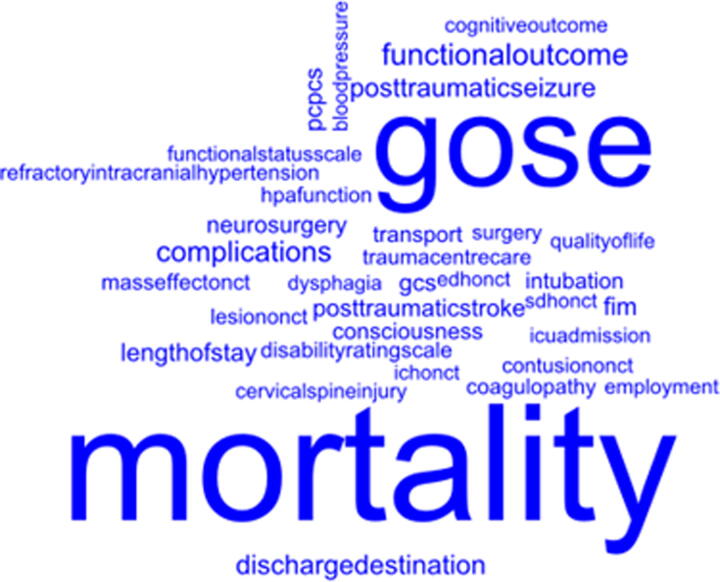
Word cloud of outcomes assessed against the clinical factors (size of word denotes frequency in included records). GCS, Glasgow Coma Scale; GOSE, Glasgow Outcome Scale-Extended; PCPCS, Paediatric Cerebral Performance Category Scale.

### AUS-TBI consensus process

AUS-TBI integrates multiple stakeholders. The contribution of (i) clinicians and researchers, (ii) people with lived experience, and (iii) people identifying as Aboriginal or Torres Strait Islander has been sought at several stages of development of the data dictionary. Improving the health of Aboriginal and Torres Strait Islander peoples is a national health priority and a priority for injury control, therefore, their engagement in this project was critically important.^12^ In this study area, clinicians and researchers were consulted to further develop the list of observed predictors toward an accurate, feasible list of prospective items for the data dictionary. The consensus group comprised 25 clinician and researcher participants from a range of backgrounds who were members of AUS-TBI and who self-nominated for this clinical study area consensus process. The consensus process was organized by two members of the Initiative Steering Committee (P.C., M.F.) and Regina Hill (R.H.). Consultation occurred in a real-time virtual meeting, facilitated by an external consultant (R.H.), and via email.

The consensus process occurred across five stages. During the first stage, participants considered the results of data extraction (at the time of the consensus meeting, data had been extracted from 125 of the 313 records included in the review). During this stage, there was also opportunity for predictors not identified in the review to be considered. Participants reviewed judgments of predictive value for each clinical factor identified in data extraction. Factors with a high (3) or medium (2) predictive value progressed to the next stage, whereas those agreed to have a low (1) or null (0) predictive value were not considered further. During stages two, three, and four, the coverage, feasibility, and implementation fidelity of the predictors were considered, respectively, as described in Gabbe et al.^10^ Briefly, coverage was assessed according to how commonly the measure was collected and its applicability to the target population. The feasibility of collection referred to how easy the measure was to complete and the frequency, cost, and timing of collection. Reliability was based on how data were collected (i.e., patient, clinician, other parties) and implementation fidelity. Comparability was determined by whether or not the clinical factor could be used in a way that allows benchmarking. The fifth stage occurred after data extraction was complete when the list of prioritized clinical factors was updated to include additional identified factors (described below) and the list recirculated to the consensus group for further input via circular email. This iterative process continued until no further disagreement was identified.

There were 29 factors extracted, or identified, during the consensus process, which were not within the scope of the *clinical review* and instead presented in other reviews in this series. Demographic factors such as age and sex were included in the social study area (series article 2) and height and weight were reported in the health review (series article 3).^10,13^ The biological area included blood gases, measures of coagulation, and imaging (series article 5).^14^ Interventions (e.g., intubation) and measures of intervention, including cerebral perfusion pressure and brain tissue oxygenation, were included in the acute interventions review (series article 6).^15^

### Differences between protocol and this iteration of the review

There were differences between the protocol registered in PROSPERO and methods used for this iteration of the review. Joanna Briggs Institute extraction tools were not used, as standardized data extraction sheets were built in Google Sheets. The methodological quality of the included studies was not assessed, as the purpose was to identify possible clinical factors that had been utilized to predict outcomes, not to assess the methodology of each study. Studies with a sample size of <100 were excluded to avoid the limitations associated with the reliability of results in studies with a smaller sample size.

## Results

The searches identified 22,441 records, including 13,613 duplicates. The title and abstract of 8828 records were screened and 7691 were excluded. The full text of 1137 studies were obtained and screened in full. Out of these, 824 records were excluded resulting in the inclusion of 313 studies in this iteration of the review (Fig. 1).

The 313 included records were published between 1986 and 2022. The majority of these records were published after 2016. The 988 raw factors were homogenized to 19 unique factors (for example, various GCS scores, GCS 3–4, GCS 5–6, GCS <9, GCS 9–12, GCS “severe,” were homogenized to “GCS”) and the 230 raw outcomes to 37 (for example, GOS 1–3, GOS 4, GOS 4–5, GOS 5, GOS >5 were homogenized to GOS) (Supplementary Data S1). This process of homogenization was carried out firstly in R (where semistructured text was coerced to factors), and the resultant unique factors were reviewed by clinicians with area expertise to ensure appropriate categorization of these raw factors.

The most frequently identified predictors were the GCS, pupillary abnormalities, and blood pressure (Fig. 2). There was variation in the definition and timing of assessment for some of the clinical factors identified, particularly factors which are dynamic and can be assessed at multiple time points (e.g., physiological measures such as blood pressure), or which have multiple components (e.g., GCS), or various methods of measurement (e.g., injury severity measures). The timings of assessment for the blood pressure included initial/first, pre-hospital, admission, emergency department (ED) admission, and lowest within 24 h of admission; the majority of measures were systolic blood pressure in mmHg. The eye, motor, verbal components of GCS and the sum GCS were extracted as clinical factors, and in this review, combined as one factor for analysis. Although the timing of injury severity measures is consistent (discharge from acute hospital), there were multiple measures/methods of measuring severity, although the majority used Abbreviated Injury Scale (AIS) or its derivatives. PTA was a significant predictor of functional and disability outcomes.

Data extraction for predictor time, or time of injury since baseline, were not mandated fields in the data extraction sheet and had a lower rate of completion than other data fields. Timing was not anticipated to be a critical data point and, thus, it was not a prescribed data field. Further detail about the predictor:outcome relationship is presented in the Supplementary Material (Supplementary Data S2).

The most frequently reported outcomes were mortality, Glasgow Outcome Score (GOS) and Glasgow Outcome Score-Extended (GOSE) (Fig. 3). Complications (measured at discharge from acute hospital) were reported as both a clinical predictor and outcome, more frequently the latter. Complications included specific conditions or events (e.g., acute kidney injury, acute lung injury, cardiac arrest) and were also reported in more general terms (e.g., in-hospital morbidity, overall complications, delayed clinical deterioration).

The data items and their prioritizations pre- and post-consensus are listed in Table 3. There were 14 clinical factors suggested by consensus group participants, which had not been identified during data extraction. Of these, 10 were excluded following the consensus process as they were either duplicative (e.g., hypertension, captured by blood pressure) or outside the scope of this review (e.g., outcome or process measures, or interventions). Four were within the scope of this review and these are presented as “new” in Table 3, and of these, two were assigned a high priority ranking: Acute Physiology and Chronic Health Evaluation (APACHE) II and venous thromboembolism (VTE) complications. APACHE II was included as it is an established predictor of intensive care unit mortality in trauma patients, which combines clinical factors included in this review (e.g., measures of hemodynamic stability, GCS) and also elements covered in the accompanying social review (age, sex) and biological review (predominantly blood biomarkers).^10,14^ VTE complications were included on the basis that it is a common and potentially fatal complication in trauma patients, including those with moderate-severe TBI. Three factors were not assigned a predictive value during the consensus meeting for the following reasons: (i) penetrating trauma emerged as an injury cause factor following completion of data extraction, (ii) New Injury Severity Score (NISS) is derived from the AIS,^54^ and (iii) heart rate variability was considered an emerging predictor and excluded on the basis that the evidence base for this measurement is still developing, and it is not currently a part of routine monitoring in critical care. To summarize, out of the 19 factors identified during data extraction, 12 were recommended for inclusion in the data dictionary following the consensus process. Of the 14 additional factors suggested by consensus group participants, 4 were within the scope of this review and 2 were recommended for inclusion and could be derived from the factors identified by the literature review. The confirmed list of prioritized predictors described those clinical factors adjudged to be accurate, feasible data items on the basis of the available literature and clinical and research expertise.

**Table 3.** Clinical Factors Identified Pre-Consensus Meeting and the Prioritization of These Factors Post-Consensus Meeting

Category	Clinical factor	Predictive value pre-consensus	Prioritization post-consensus^*f*^	Timing of data collection	Recommendation for inclusion in data dictionary
Injury severity/type/associated injury	Cervical fracture^a^	Medium	Low	At discharge from acute hospital	N
	Isolated TBI vs. TBI with associated extracranial injury^b^	Null	Low	At discharge from acute hospital	N
	Multiple trauma	Null	**High**	At discharge from acute hospital	Y (derived from AIS)
	Number of systems injured^a^	Low	Low	At discharge from acute hospital	N
	Penetrating trauma	Medium	^b^	Initial assessment	Y
	ASCOT	Low	**Low**	On admission and AIS on discharge	N
	AIS	Medium	**High**	At discharge from acute hospital	Y
	ISS^a^	Low	**Low**	At discharge from acute hospital	N
	Injury severity/type^a^	Low	**Low**	At discharge from acute hospital	N
	NISS^a^	^b^	^b^	At discharge from acute hospital	N
	PIM 2	Low	**Low**	PICU admission	N
	PRISM	Low	**Low**	Within first four hours of PICU admission	N
	PTS	Low	**Low**	Initial assessment	N
	RTS	Low	**Low**	Initial assessment	N
	TRISS	Low	**Low**	At discharge from acute hospital	N
Complications	Acute kidney injury	Low	Low	At discharge from acute hospital	N
	Acute lung injury	Low	Low	At discharge from acute hospital	N
	Acute respiratory distress syndrome	Medium	Low	At discharge from acute hospital	N
	Hospital acquired pneumonia	Medium	Low	At discharge from acute hospital	N
	Post-traumatic seizures	Medium	**Medium**	At discharge from acute hospital	Y
	Sepsis	Low	**Low**	At discharge from acute hospital	N
	Sequential Organ Failure Score >5	Low	**Low**	At discharge from acute hospital	N
	VTE complications	^b^	**High**	At discharge from acute hospital	Y
GCS/derivatives	GCS	High	**High**	Admission^d^	Y
	GCS eye response	Low	Low	On admission^e^	Y
	GCS motor response	Low	Low	On admission^e^	N
	Loss of consciousness	Null	Null	Initial assessment	
PTA	PTA duration	Low	**Medium/high**	On recovery	Y
Physiological-hemodynamic stability	APACHE II^c^	^b^	**High**	Within 24 h of ICU admission	Y
	Body temperature	Low	**Low**	On admission	Y
	Hypoxemia	Low	**High**	On admission	Y
	Respiratory rate	Null	**Low**	On admission	N
	Blood pressure	High	**High**	On admission	Y
	Heart rate	Low	**High**	On admission	Y
	Heart rate variability	b	b	ICU stay	N
	Shock index	Low	**High**	On admission	Y (derived from HR and BP)
Pupils	Pupil reactivity	High	**High**	On admission	Y
	Pupil size/shape	Medium	Low	On admission	N

^a^ Not recommended for inclusion in the data dictionary but can be derived from AIS.

^b^ Clinical factors identified during consensus meeting, did not have predictive value prior to meeting.

^c^APACHE II is used in ICU patients.

^d^ If not valid or available at admission, impute from pre-intubation value.

^e^ If not valid or available at admission, impute from pre-hospital value.

^f^ Black text denotes a null finding, orange denotes low rating, blue denotes medium rating, and green denotes high rating of predictive value based on the findings of the systematic review.

TBI, traumatic brain injury; AIS, Abbreviated Injury Scale; ASCOT, A Severity Characterisation of Trauma; ISS, Injury Severity Score; NISS, New Injury Severity Score; PIM 2, Pediatric Index of Mortality 2; PRISM, Paediatric Risk of Mortality Score; PTS, Pediatric Trauma Score; RTS, Revised Trauma Score; TRISS, Trauma Score and Injury Severity Score; VTE, venous thromboembolism; GCS, Glasgow Coma Scale; PTA, post-traumatic amnesia; APACHE, Acute Physiology and Chronic Health Evaluation; ICU, intensive care unit; HR, heart rate, BP, blood pressure.

## Discussion

During this review, there were a small number of consistently reported factors that had a strong predictive value for mortality and disability as measured by the GOSE and GOS. These factors were predominantly measures of consciousness—as assessed by the GCS and its subcomponents—and physiological responses to injury, such as pupillary response and standard hemodynamic measures. The total GCS was included as a high-priority factor as it captures the subscales (GCS eye, verbal, and motor). Other factors with high predictive value that were identified included the AIS (which describes the type, location, and severity of injury) and the presence of multiple trauma. These factors have been included in prognostic models with a high degree of certainty.^55,56^ Complications were identified as both clinical factors and clinical outcomes, for example, acute kidney injury was studied as a clinical factor associated with the outcomes of mortality and GOSE, and also as a clinical outcome associated with GCS scores.

Injury severity was associated with both disability and mortality. We identified various ways of defining injury severity; the majority were anatomical, physiological, or combined (i.e., anatomical and physiological) scoring systems. Through the consensus process, the AIS was identified as having the highest value of all the identified injury severity predictors—based on its predictive value, common use, high comparability, and high reliability. Other measures of injury severity (for example, the Injury Severity Score [ISS] and the NISS) can be derived from the AIS, and the AIS also captures other injury type and associated injury factors, such as multiple trauma, the number of systems injured, and isolated TBI or TBI with associated extracranial injury. Head AIS is specific to TBI, which is another advantage; other measures such as the ISS provide the total injury score for the whole body and, therefore, are considered less likely to predict TBI outcomes. The components of the AIS specified for inclusion in the final data dictionary were AIS skull fracture, high spinal injury, chest injury, and orthopedic injury. It is important to note that injury type and severity are also, and perhaps better, assessed through imaging, for example, the Marshall CT score (see series article 5).^14^

The GCS was most frequently associated with the clinical outcomes of GOSE and mortality, with lower GCS scores associated with poorer outcomes. There was inconsistency in the use of the GCS in terms of the timing of assessment. In this review, we identified several time-points at which GCS was assessed, including pre-hospital, at admission, and the best score within 24 h of admission.^57–59^ The timing of assessment can influence the predictive ability of the GCS. The GCS at hospital admission has been shown to have a stronger association with outcome compared with the pre-hospital GCS, however, the GCS at admission may be affected by pre-hospital interventions including sedation, analgesia, neuromuscular blockade, and intubation.^30,60,61^

We also identified variation in the component of the GCS score reported, with some studies using only GCS motor scores rather than the total GCS score as a predictor of outcomes.^30,59^ In severe TBI, the motor component of the GCS has been identified as containing the same prognostic information as the total GCS score.^30^ Lenartova et al. (2007) found that using only the motor response of the GCS had similar ability as the full GCS score to predict mortality at 6 months.^62^ A systematic review by Reith et al. (2015) found that the reliability of the GCS assessment is higher for each of the separate components, rather than the derived sum score.^63^ The authors suggested that this might be owing to the sum score requiring each component to be assessed and then combined—introducing four sources of potential variation—and also that the sum score has more possible scoring options.

In TBI, abnormalities in pupillary response or pupil size are associated with neurological deterioration and secondary brain injury and are predictive of poor outcomes.^64^ Assessment of pupillary reactivity is a standard procedure in neurological evaluation, and pupil reactivity is a core component of established TBI prediction models.^25,50,60^ In this review, pupillary abnormalities were identified as having high prognostic value and were associated with mortality and poorer functional outcomes as assessed by the GOSE.

There was variation in the way pupil abnormalities were reported in the included studies, in terms of both the terminology used and the timing of the assessment. This included fixed pupillary response on hospital admission, absent pupillary reflexes on admission, pupil reactivity on arrival in the ED, or the values at the scene of the accident were for patients arriving intubated.^27,28,32^ Majdan et al. (2015) assessed pupillary reactivity in the field compared with hospital admission and found that it had better performance in predicting 6-month mortality when it was assessed at admission.^30^

The subjective nature of pupillary assessment and low interrater reliability should also be considered when assessing its prognostic value. Previous studies have reported limited interrater reliability for the size, shape, and reactivity scores among diverse practitioners performing manual pupillary assessment.^64^,^65^ The use of objective pupillary evaluation using automated pupillary assessments would standardize the assessment of pupillary function and provide higher reliability.^26^

Blood pressure was found to have high predictive value for mortality and GOSE. Hypotension in the pre-hospital or early hospital phase of TBI management is associated with increased risk of mortality, however, the thresholds for “low” blood pressure and shock index vary.^66–71^ In the studies included in this review, cut-off points to define hypotension were defined inconsistently, although <90 mmHg was commonly used. There was also variation in the way blood pressure was reported, with systolic, diastolic, and mean arterial pressures all reported. The timing of blood pressure readings reported included pre-hospital, admission, and the lowest measurements in the time period (e.g., 12 h) since admission. There is some evidence that the threshold for hypotension in patients with moderate to severe TBI should be raised, redefined, and modified by age groups.,^71,72^ In a study investigating pre-hospital blood pressure and mortality, a linear relationship was found between lowest pre-hospital systolic blood pressure and mortality across a wide range.^66^

It is important to note that there are clinical characteristics commonly measured to assess patients and predict outcomes following discharge from acute hospital admission, particularly in the rehabilitation setting, which were not identified during this review. The focus of this review was on early prediction of outcomes during the acute care phase, not the rehabilitation setting. Of note, PTA and duration of PTA, assessed after the acute phase of care, were reported as highly prognostic for functional and disability outcomes in survivors of moderate-severe TBI.^72–75^[Bibr B73] Despite the search being limited to acute care settings, PTA was still identified during this review and following the consensus process was considered important to collect and included as a medium/high-value factor.

### Strengths and limitations

The key strengths of this systematic review were the comprehensive search strategy and the breadth of clinical factors included as predictors of any clinical outcome. The search strategy included eight databases, articles were screened by at least two independent reviewers, and inclusion and exclusion criteria were consistently applied to ensure that only studies examining the relevant patient population (moderate to severe TBI) in the appropriate study setting (acute care) were included. A broad range of clinical factors were identified as being associated with clinical outcomes, including physiological measurements, injury-related factors, complications, and measures of consciousness.

There are several limitations of this study. There was great variation in the way clinical outcomes were defined and measured across the available publications; given this heterogeneity, a meta-analysis was not feasible. Data extraction was unable to be completed prior to the consensus meeting, which meant not all factors could be reviewed during this initial consensus process. The focus on prediction in the initial phase of care meant that clinical factors assessed at time points beyond the acute care phase, such as in rehabilitation settings, were not captured by this review. The lack of long-term outcomes and patient-reported outcomes may also be a result of limiting this review to the acute care setting, in which the survival element of treatment is emphasized, rather than considering functional disability outcomes.

## Conclusion

During this systematic review, 313 records of studies evaluating associations between clinical factors and clinical outcomes in patients with moderate-severe TBI were identified. A small number of factors with high predictive value were reported consistently, and these were included in the final data dictionary, however, there was a lack of standardization in data collection for these factors across studies, including variation in the timing of assessments (e.g., pre-hospital vs. admission), the type of measure used (e.g., systolic vs. diastolic blood pressure vs. pulse pressure) or the component of measure used (GCS motor vs. total GCS score). Variation limits the ability to directly compare data elements, and to combine databases to link acute and longitudinal data and highlights the importance of using clearly defined and standardized data elements in the design of the AUS-TBI single-data dictionary.^76^ The findings of this review and the subsequent consensus process have informed the development of an evidence-informed data dictionary for moderate-severe TBI in Australia, which may be useful for predicting outcomes in Australian patients (and potentially internationally), and which could also be used to evaluate the efficacy of interventions in clinical trials.^7^ Such a resource will be useful for developing benchmarks in TBI care, evaluating hospital and system outcomes, facilitating research, and determining trends in TBI care.

## The AUS-TBI Investigators

**Group Authorship:** The following AUS-TBI Investigators should be indexed with their individual full names as “Collaborators” in the National Library of Medicine PubMed database and other repositories. Thank you.

The AUS-TBI investigators are as follows:

*Tara Alexander* (ORCID 0000-0001-5234-7821) (Australasian Rehabilitation Outcomes Centre & Australian Health Services Research Institute, Faculty of Business and Law, University of Wollongong, Wollongong, New South Wales, Australia);

*Vicki Anderson* (Psychology Service, The Royal Children’s Hospital, Melbourne, Victoria, Australia; Clinical Sciences Research, Murdoch Children’s Research Institute, Melbourne, Victoria, Australia);

*Ana Antonic-Baker* (Department of Neuroscience, Central Clinical School, Monash University, Melbourne, Victoria, Australia);

*Elizabeth Armstrong* (School of Medical and Health Sciences, Edith Cowan University, Perth, Western Australia, Australia);

*Franz E Babl* (Department of Emergency Medicine, The Royal Children’s Hospital, Melbourne, Victoria, Australia; Departments of Paediatrics and Critical Care, University of Melbourne, Melbourne, Victoria, Australia; Murdoch Children’s Research Institute, Melbourne, Victoria, Australia);

*Matthew K Bagg* (ORCID 0000-0002-4812-3814) (Curtin Health Innovation Research Institute, Faculty of Health Sciences, Curtin University, Perth, Western Australia, Australia; School of Health Sciences, University of Notre Dame Australia, Perth, Western Australia, Australia; Perron Institute for Neurological and Translational Science, Perth, Western Australia, Australia; Centre for Pain IMPACT, Neuroscience Research Australia, Sydney, New South Wales, Australia);

*Zsolt J Balogh* (Department of Traumatology, John Hunter Hospital and University of Newcastle, Newcastle, New South Wales, Australia);

*Karen M Barlow* (Acquired Brain Injury in Children Research Program, Queensland Children’s Hospital, Brisbane, Queensland, Australia; Centre for Children’s Health Research, University of Queensland, Brisbane, Queensland, Australia);

*Judith Bellapart* (Department of Intensive Care Services, Royal Brisbane and Women’s Hospital, Brisbane, Queensland, Australia; Faculty of Medicine, University of Queensland, Brisbane, Queensland, Australia);

*Niranjan Bidargaddi* (Flinders Digital Health Centre, College of Medicine & Public Health, Flinders University, Adelaide, South Australia, Australia);

*Erika Bosio* (Centre for Clinical Research in Emergency Medicine, Harry Perkins Institute of Medical Research, Perth, Western Australia, Australia; School of Biomedical Science & School of Medicine, University of Western Australia, Perth, Western Australia, Australia);

*Peter Bragge* (BehaviourWorks Australia, Monash Sustainable Development Institute, Monash University, Melbourne, Victoria, Australia);

*Michael Bynevelt* (School of Surgery, The University of Western Australia, Perth, Western Australia, Australia; Neurological Intervention and Imaging Service of Western Australia, Sir Charles Gairdner Hospital, Perth, Western Australia, Australia);

*Karen Caeyenberghs* (Cognitive Neuroscience Unit, School of Psychology, Deakin University, Geelong, Victoria, Australia);

*Peter A Cameron* (National Trauma Research Institute, Melbourne, Victoria, Australia; School of Public Health and Preventive Medicine, Monash University, Melbourne, Victoria, Australia; Emergency and Trauma Centre, The Alfred Hospital, Melbourne, Victoria, Australia);

*Jacquelin Capell* (Australasian Rehabilitation Outcomes Centre & Australian Health Services Research Institute, Faculty of Business and Law, University of Wollongong, Wollongong, New South Wales, Australia);

*Kevin E K Chai* (School of Population Health, Faculty of Health Sciences, Curtin University, Perth, Western Australia, Australia; Curtin Institute for Computation, Curtin University, Perth, Western Australia, Australia);

*Lyndsey E Collins-Praino* (School of Biomedicine, University of Adelaide, Adelaide, South Australia, Australia);

*D J Jamie Cooper* (Australian and New Zealand Intensive Care Research Centre, School of Public Health and Preventive Medicine, Monash University, Melbourne, Victoria, Australia; Department of Intensive Care and Hyperbaric Medicine, The Alfred, Melbourne, Victoria, Australia);

*Gill Cowen* (School of Medicine, Faculty of Health Sciences, Curtin University, Perth, Western Australia, Australia);

*Louise M Crowe* (Clinical Sciences Research, Murdoch Children’s Research Institute, Melbourne, Victoria, Australia; Department of Paediatrics, University of Melbourne, Melbourne, Victoria, Australia);

*Tim Cudmore* (AUS-TBI Lived Experience Advisory Group);

*Jennifer Cullen* (Synapse, Brisbane, Queensland, Australia; James Cook University, Townsville, Queensland, Australia; Menzies Health Institute Queensland, Griffith University, Brisbane, Queensland, Australia);

*Kate Curtis* (ORCID 0000-0002-3746-0348) (Susan Wakil School of Nursing and Midwifery, Faculty of Medicine and Health, The University of Sydney, New South Wales, Australia; Illawarra Shoalhaven Local Health District, Wollongong, New South Wales, Australia; Illawarra Health and Medical Research Institute, Wollongong, New South Wales, Australia; George Institute for Global Health, Sydney, New South Wales, Australia);

*Anthony Delaney* (Division of Critical Care, The George Institute for Global Health, Sydney, New South Wales, Australia; Malcolm Fisher Department of Intensive Care Medicine, Royal North Shore Hospital, Sydney, New South Wales, Australia; Northern Clinical School, Sydney Medical School, University of Sydney, Sydney, New South Wales, Australia; Australian and New Zealand Intensive Care Research Centre, School of Public Health and Preventive Medicine, Monash University, Melbourne, Victoria, Australia);

*Graeme Dibdin* (AUS-TBI Lived Experience Advisory Group);

*Sandra Eades* (Centre for Epidemiology and Biostatistics, Melbourne School of Population and Global Health, University of Melbourne, Melbourne, Victoria, Australia; School of Medicine, Faculty of Health Sciences, Curtin University, Perth, Western Australia, Australia);

*Gary F Egan* (Monash Biomedical Imaging & School of Psychological Sciences, Monash University, Melbourne, Victoria, Australia);

*Daniel Y Ellis* (Department of Trauma, Royal Adelaide Hospital, Adelaide, South Australia, Australia; Statewide South Australian Trauma Service, South Australia, Australia; School of Public Health and Tropical Medicine, James Cook University, Queensland, Australia);

*Ari Ercole* (Division of Anaesthesia, University of Cambridge, Addenbrooke’s Hospital, Cambridge, United Kingdom; Cambridge Centre for AI in Medicine, University of Cambridge, United Kingdom);

*Daniel M Fatovich* (Emergency Medicine, Royal Perth Hospital, University of Western Australia, Perth, Western Australia, Australia; Centre for Clinical Research in Emergency Medicine, Harry Perkins Institute of Medical Research, Perth, Western Australia, Australia);

*Murray J Fisher* (Susan Wakil School of Nursing and Midwifery, Faculty of Medicine and Health, University of Sydney, New South Wales, Australia; Royal Rehab, Ryde, Sydney, New South Wales, Australia);

*Mark Fitzgerald* (National Trauma Research Institute, Melbourne, Victoria, Australia);

*Melinda Fitzgerald* (Curtin Health Innovation Research Institute, Faculty of Health Sciences, Curtin University, Perth, Western Australia, Australia; Perron Institute for Neurological and Translational Science, Perth, Western Australia, Australia);

*Jennifer Fleming* (School of Health and Rehabilitation Sciences, The University of Queensland, Brisbane, Queensland, Australia);

*Roslyn Francis* (Department of Health, Government of Western Australia, Perth, Western Australia, Australia);

*Belinda J Gabbe* (School of Public Health and Preventive Medicine, Monash University, Melbourne, Victoria, Australia; Health Data Research UK, Swansea University Medical School, Swansea University, Singleton Park, United Kingdom);

*Adelle Gadowski* (School of Public Health and Preventive Medicine, Monash University, Melbourne, Victoria, Australia);

*John Gilroy* (Aboriginal and Torres Strait Islander Research, Faculty of Medicine and Health, University of Sydney, Sydney, New South Wales, Australia);

*Mitchell A Hansen* (Department of Neurosurgery, John Hunter Hospitals and University of Newcastle, Newcastle, New South Wales, Australia);

*James E Harrison* (College of Medicine and Public Health, Flinders University, Adelaide, South Australia, Australia);

*Luke J Haseler* (Curtin Health Innovation Research Institute, Faculty of Health Sciences, Curtin University, Perth, Western Australia, Australia);

*Leanne Hassett* (Institute for Musculoskeletal Health & Sydney School of Health Sciences, Faculty of Medicine and Health, University of Sydney, Sydney, New South Wales, Australia; Sydney Local Health District, Sydney, New South Wales, Australia);

*Sarah C Hellewell* (Curtin Health Innovation Research Institute & School of Medicine, Faculty of Health Sciences, Curtin University, Perth, Western Australia, Australia; Perron Institute for Neurological and Translational Science, Nedlands, Western Australia, Australia);

*Amelia J Hicks* (School of Psychological Sciences, Monash University, Melbourne, Victoria, Australia; Monash Epworth Rehabilitation Research Centre, Epworth Healthcare, Melbourne, Victoria, Australia; Brain Injury Research Center, Icahn School of Medicine at Mount Sinai, New York City, New York, United States of America);

*Andrew F Hill* (College of Science, Health and Engineering, La Trobe University, Melbourne, Victoria, Australia);

*Andrew J A Holland* (ORCID 0000-0003-3745-8704) (The Children’s Hospital at Westmead Clinical School, Faculty of Medicine and Health, University of Sydney, Sydney, New South Wales, Australia);

*Stephen Honeybul* (Department of Neurosurgery, Sir Charles Gairdner Hospital, Perth, Western Australia, Australia; Department of Neurosurgery, Royal Perth Hospital, Perth, Western Australia, Australia);

*Rosalind L Jeffree* (Kenneth G. Jamieson Department of Neurosurgery, Royal Brisbane and Women’s Hospital, Brisbane, Queensland, Australia; Royal Brisbane Clinical School, School of Medicine, University of Queensland, Brisbane, Queensland, Australia);

*Chris Joyce* (Intensive Care Unit, Princess Alexandra Hospital, Brisbane, Queensland, Australia; School of Medicine, University of Queensland, Brisbane, Queensland, Australia);

*Elizabeth Kendall* (Menzies Health Institute Queensland, Griffith University, Brisbane, Queensland, Australia);

*Kate King* (John Hunter Trauma Service, John Hunter Hospital, Newcastle, New South Wales, Australia; College of Health, Medicine and Wellbeing, University of Newcastle, Newcastle, New South Wales, Australia);

*Natasha A Lannin* (Department of Neuroscience, Central Clinical School, Monash University, Melbourne, Victoria, Australia; Alfred Health, Melbourne, Victoria, Australia);

*Meng Law* (Departments of Neuroscience and Radiology, Monash University, Melbourne, Victoria, Australia; Alfred Health, Melbourne, Victoria, Australia; Alzheimer’s Disease Research Center & Department of Neurological Surgery, Keck School of Medicine, University of Southern California, Los Angeles, California, United States of America);

*Andrew I R Maas* (Department of Neurosurgery, Antwerp University Hospital and University of Antwerp, Edegem, Belgium);

*Adam Mahoney* (Trauma Service, Royal Hobart Hospital, Hobart, Tasmania, Australia; 2nd General Health Battalion, Australian Defence Force);

*Peter Makin* (AUS-TBI Lived Experience Advisory Group);

*Peter Mayhew* (AUS-TBI Lived Experience Advisory Group);

*Alison McDonald* (AUS-TBI Lived Experience Advisory Group);

*Skye McDonald* (School of Psychology, University of New South Wales, Sydney, New South Wales, Australia);

*Stuart J McDonald* (Department of Neuroscience, Central Clinical School, Monash University, Melbourne, Victoria, Australia);

*Ancelin McKimmie* (School of Public Health and Preventive Medicine, Monash University, Melbourne, Victoria, Australia);

*Robert McNamara* (Department of Intensive Care Medicine, Royal Perth Hospital, Perth, Western Australia, Australia; School of Medicine, Faculty of Health Sciences, Curtin University, Perth, Western Australia, Australia);

*Shiv Meka* (Department of Health, Government of Western Australia, Perth, Western Australia, Australia);

*David K Menon* (Division of Anaesthesia, University of Cambridge, Addenbrooke’s Hospital, Cambridge, United Kingdom; Wolfson Brain Imaging Centre, University of Cambridge, Cambridge, United Kingdom);

*Gary Mitchell* (Emergency and Trauma Unit, Royal Brisbane and Women’s Hospital, Brisbane, Queensland, Australia; Royal Brisbane Clinical Unit, University of Queensland, Brisbane, Queensland, Australia; Jamieson Trauma Institute, Brisbane, Queensland, Australia; Queensland Rugby Union, Brisbane, Queensland, Australia);

*Rowena Mobbs* (Brain & Mind Centre, University of Sydney, Sydney, New South Wales, Australia; Macquarie University, Sydney, New South Wales, Australia);

*Fatima A Nasrallah* (Queensland Brain Institute, University of Queensland, Brisbane, Queensland, Australia);

*Virginia F J Newcombe* (PACE Section, Department of Medicine, Addenbrooke’s Hospital, University of Cambridge, Cambridge, United Kingdom);

*Terence J O’Brien* (Department of Neuroscience, Central Clinical School, Monash University, Melbourne, Victoria, Australia);

*John H Olver* (Epworth Healthcare, Melbourne, Victoria, Australia; Department of Medicine, Monash University, Melbourne, Victoria, Australia);

*Gerard M O’Reilly* (National Trauma Research Institute, Melbourne, Victoria, Australia; Emergency and Trauma Centre, The Alfred Hospital, Melbourne, Victoria, Australia; School of Public Health and Preventive Medicine, Monash University, Melbourne, Victoria, Australia);

*Tamara Ownsworth* (The Hopkins Centre, Menzies Health Institute Queensland, Griffith University, Brisbane, Queensland, Australia; School of Applied Psychology, Griffith University, Brisbane, Queensland, Australia);

*Paul M Parizel* (University of Antwerp, Edegem, Belgium; Department of Radiology, Royal Perth Hospital & University of Western Australia, Perth, Western Australia, Australia; West Australian National Imaging Facility Node, Perth, Western Australia, Australia);

*Michael Parr* (Intensive Care Unit, Liverpool Hospital, University of New South Wales, Sydney, New South Wales, Australia; Intensive Care Unit, Macquarie University Hospital, Macquarie University, Sydney, New South Wales, Australia);

*Jennie L Ponsford* (School of Psychological Sciences, Monash University, Melbourne, Victoria, Australia; Monash Epworth Rehabilitation Research Centre, Epworth Healthcare, Melbourne, Victoria, Australia);

*Bruce Powell* (AUS-TBI Lived Experience Advisory Group);

*Patricia Ratajczak* (AUS-TBI Lived Experience Advisory Group);

*Michael C Reade* (Faculty of Medicine, University of Queensland, Royal Brisbane and Women’s Hospital, Brisbane, Queensland, Australia; Joint Health Command, Australian Defence Force, Canberra, Australian Capital Territory, Australia);

*Sandy Reeder* (Department of Neuroscience, Central Clinical School, Monash University, Melbourne, Victoria, Australia; Department of Epidemiology and Preventive Medicine, School of Public Health and Preventive Medicine, Monash University, Melbourne, Victoria, Australia);

*Christopher Reid* (School of Public Health, Faculty of Health Sciences, Curtin University, Perth, Western Australia, Australia; Department of Epidemiology and Preventive Medicine, School of Public Health and Preventive Medicine, Monash University, Melbourne, Victoria, Australia);

*Julia Robertson* (AUS-TBI Lived Experience Advisory Group);

*Suzanne Robinson* (School of Population Health, Faculty of Health Sciences, Curtin University, Perth, Western Australia, Australia);

*Danette Rowse* (AUS-TBI Lived Experience Advisory Group);

*Stephen E Rose* (The Australian e-Health Research Centre, Commonwealth Scientific and Industrial Research Organisation, Brisbane, Queensland, Australia);

*Jeffrey V Rosenfeld* (Department of Neurosurgery, The Alfred Hospital, Melbourne, Victoria, Australia; Department of Surgery, Monash University, Melbourne, Victoria, Australia; F. Edward Hébert School of Medicine, Uniformed Services University of the Health Sciences, Bethesda, Maryland, United States of America);

*Jason P Ross* (Molecular Diagnostic Solutions, Health and Biosecurity, Commonwealth Scientific and Industrial Research Organisation, Australia);

*Nick Rushworth* (Brain Injury Australia, Sydney, New South Wales, Australia);

*Adam Scheinberg* (Neurodevelopment and Rehabilitation Research, Murdoch Children’s Research Institute, Melbourne, Victoria, Australia; Department of Paediatrics, University of Melbourne, Melbourne, Victoria, Australia);

*Bridgette D Semple* (Department of Neuroscience, Central Clinical School, Monash University, Melbourne, Victoria, Australia; Alfred Health, Melbourne, Victoria, Australia; Department of Medicine, Royal Melbourne Hospital, The University of Melbourne, Melbourne, Victoria, Australia);

*Sandy R Shultz* (Department of Neuroscience, Central Clinical School, Monash University, Melbourne, Victoria, Australia);

*Grahame K Simpson* (Brain Injury Rehabilitation Research Group, Ingham Institute for Applied Medical Research, Sydney, New South Wales, Australia; John Walsh Centre for Rehabilitation Research, Sydney School of Medicine, University of Sydney, Sydney, New South Wales, Australia);

*Warwick J Teague* (ORCID 0000-0003-4747-6025) (Trauma Service & Department of Paediatric Surgery, The Royal Children’s Hospital, Melbourne, Victoria, Australia; Surgical Research, Murdoch Children’s Research Institute, Melbourne, Victoria, Australia; Department of Paediatrics, University of Melbourne, Melbourne, Victoria, Australia);

*Leanne Togher* (Speech Pathology, School of Health Sciences, Faculty of Medicine and Health, University of Sydney, Sydney, New South Wales, Australia);

*Andrew A Udy* (Australian and New Zealand Intensive Care Research Centre, School of Public Health and Preventive Medicine, Monash University, Melbourne, Victoria, Australia; Department of Intensive Care and Hyperbaric Medicine, The Alfred Hospital, Melbourne, Victoria, Australia);

*Kirsten Vallmuur* (Centre for Healthcare Transformation, Australian Centre for Health Services Innovation, Queensland University of Technology, Brisbane, Queensland, Australia; Jamieson Trauma Institute, Brisbane, Queensland, Australia);

*Dinesh Varma* (Department of Radiology, The Alfred Hospital, Melbourne, Victoria, Australia; Department of Surgery, Monash University, Melbourne, Victoria, Australia; National Trauma Research Institute, Melbourne, Victoria, Australia);

*James Vickers* (Wicking Dementia Research and Education Centre, College of Health and Medicine, University of Tasmania, Hobart, Tasmania, Australia);

*Janet Wagland* (Brightwater Group, Perth, Western Australia, Australia);

*James Walsham* (Intensive Care Unit, Princess Alexandra Hospital, Brisbane, Queensland, Australia; School of Medicine, University of Queensland, Brisbane, Queensland, Australia);

*Adam J Wells* (Department of Neurosurgery, Adelaide Hospital, Adelaide, South Australia, Australia; Department of Surgery, University of Adelaide, Adelaide, South Australia, Australia; Neurosurgical Research Foundation, Adelaide, South Australia, Australia);

*Luke Whiley* (Health Futures Institute, Murdoch University, Perth, Western Australia, Australia; Perron Institute for Neurological and Translational Science, Perth, Western Australia, Australia);

*Gavin Williams* (Department of Physiotherapy, Epworth Healthcare, Melbourne, Victoria, Australia; Department of Physiotherapy, University of Melbourne, Melbourne, Victoria, Australia);

*Jodie K Williams* (National Critical Care and Trauma Response Centre, Royal Darwin Hospital, Darwin, Northern Territory, Australia);

*Roslind Witham* (AUS-TBI Lived Experience Advisory Group);

*David K Wright* (Department of Neuroscience, Central Clinical School, Monash University, Melbourne, Victoria, Australia);

*Louise York* (Australian Institute of Health and Welfare, Canberra, Australian Capital Territory, Australia);

*Jesse T Young* (ORCID 0000-0001-5702-372X) (Centre for Health Equity, Melbourne School of Population and Global Health, The University of Melbourne, Melbourne, Victoria, Australia; Centre for Adolescent Health, Murdoch Children’s Research Institute, Melbourne, Victoria, Australia; School of Population and Global Health, University of Western Australia, Perth, Western Australia, Australia; National Drug Research Institute, Curtin University, Perth, Western Australia, Australia);

*Heidi Zeeman* (The Hopkins Centre, Menzies Health Institute Queensland, Griffith University, Brisbane, Queensland, Australia).

## Supplementary Material

Supplementary Data S1

## Supplementary Material

Supplementary Data S2

## Abbreviations Used

AIS: Abbreviated Injury Scale
APACHE: Acute Physiology and Chronic Health Evaluation
ASCOT: A Severity Characterisation of Trauma
AUS-TBI: The Australian Traumatic Brain Injury Initiative
BP: Blood Pressure
CT: computed tomography
DRS: Disability Rating Scale
ED: emergency department
EDH: epidural hematoma
FIM: Functional Independence Measure
GCS: Glasgow Coma Scale
GOSE: Glasgow Outcome Scale-Extended
HPA: hypothalamic pituitary adrenal axis
HR: heart rate
ICH: intracerebral hemorrhage
ICU: intensive care unit
ISS: Injury Severity Score
LOS: length of stay
NISS: New Injury Severity Score
PCPCS: Pediatric Cerebral Performance Category Scale
PICU: paediatric intensive care unit
PIM2: Paediatric Index of Mortality 2
PRISM: Pediatric Risk of Mortality Score
PTA: post-traumatic amnesia
PTS: pediatric trauma score
QoL: quality of life
RTS: Revised Trauma Score
SAPS: Simplified Acute Physiology Score
SDH: subdural hematoma
SOFS: Sequential Organ Failure Score
TBI: traumatic brain injury
TRISS: Trauma Score and Injury Severity Score
VTE: venous thrombo embolism
