# Supplementary material for: The Australian Traumatic Brain Injury Initiative: Systematic Review of Clinical Factors Associated with Outcomes in People with Moderate-Severe Traumatic Brain Injury
Source: Neurotrauma Rep. 2024 Jul 4;5(1):0. doi: 10.1089/neur.2023.0111 (PMC11286001; doi:10.1089/neur.2023.0111)
Supplement: Supplementary Data S1 [file neur.2023.0111_supplementarydatas1.docx]

Supplementary Material

In accompaniment of McKimmie et al. (2023, submitted) “The Australian Traumatic Brain Injury Initiative: systematic review of clinical factors associated with outcomes in people with moderate-severe traumatic brain injury”.

Comprising:

1. Meta-data and search strategies (this document)

2. Paper 4_AUS-TBI_Supplementary file 2_dataset_25aug23.csv

3. Paper 4_AUS-TBI_Supplementary file 3_Investigators names and affiliations.xlsx

###### **CENTRAL = 118**

Database(s): **EBM Reviews - Cochrane Central Register of Controlled Trials**January 2022
Search Strategy:

| **#** | **Searches** | **Results** |
| --- | --- | --- |
| 1 | craniocerebral trauma/ or brain injuries/ or brain hemorrhage, traumatic/ or brain stem hemorrhage, traumatic/ or cerebral hemorrhage, traumatic/ or brain injuries, diffuse/ or diffuse axonal injury/ or brain injuries, traumatic/ or brain concussion/ or brain contusion/ or chronic traumatic encephalopathy/ or brain injury, chronic/ or pneumocephalus/ or coma, post-head injury/ or head injuries, closed/ or contrecoup injury/ or post-concussion syndrome/ or head injuries, penetrating/ or intracranial hemorrhage, traumatic/ or subarachnoid hemorrhage, traumatic/ or skull fracture, depressed/ | 2999 |
| 2 | [((Brain* or Cerebr* or Cerebel* or Cortex or Crani* or Diffuse Axonal or Head or Hemisphere* or Frontal Lobe* or Temporal Lobe* or Occipital Lobe* or Parietal Lobe* or intracranial*) adj1 (Injur* or Trauma*)).mp,kf,kw,hw.] | 0 |
| 3 | [(((Posttraumatic or Traumatic) adj (brain* or encephalopath* or intracrani* or cerebel* or cerebr* or craniocerebr*)) or ((traumatic or posttraumatic) adj (brainstem or bulbar or medullary or midbrain or pontine or subarachnoid or subdural) adj (h?em?or*ag* or h?ematoma*))).mp,kf,kw,hw.] | 0 |
| 4 | [(Concuss* or Postconcuss* or Pneumocephalus or Crush* Skull* or brain commotion? or Commotio Cerebri or Contusio Cerebri or posttraumatic coma* or post-traumatic coma* or depressed skull fracture* or ((Brain* or Cerebr* or Cerebel* or Diffuse Axonal or intracrani*) adj (damage* or wound* or lacerat* or contusion?))).mp,kf,kw,hw.] | 0 |
| 5 | [((penetrat* adj3 skull* adj3 (trauma* or injur* or fracture*)) or (penetrat* adj3 head* adj3 (wound* or fracture*))).mp,kf,kw,hw.] | 0 |
| 6 | [(((severe or moderate or mild) adj GCS) or (GCS score adj3 ("3" or "4" or "5" or "6" or "7" or "8" or "9" or "10" or "11" or "12")) or ((glasgow coma scale* or glasgow outcome scale or glasgow scale* or Rancho Los Amigos) adj ("3" or "4" or "5" or "6" or "7" or "8" or "9" or "10" or "11" or "12"))).mp,kf,kw,hw.] | 0 |
| 7 | [(traumatic lesion* adj3 (Brain* or Cerebr* or Cerebel* or Cortex or Crani* or Diffuse Axonal or Head or Hemisphere* or Frontal Lobe* or Temporal Lobe* or Occipital Lobe* or Parietal Lobe* or intracranial* or encephalopath* or bulbar or medullary or midbrain or pontine or subarachnoid or subdural)).mp,kf,kw,hw.] | 0 |
| 8 | 1 or 2 or 3 or 4 or 5 or 6 or 7 | 2999 |
| 9 | vital signs/ or blood pressure/ or heart rate/ | 36116 |
| 10 | (physiological observation? or clinical observation? or (cardiac rate? or heart rate? or heartbeat? or pulse rate? or vital sign?) or (blood pressure or diastolic pressure or pulse pressure or systolic pressure)).mp. | 153251 |
| 11 | oximetry/ or hypoxia/ | 2670 |
| 12 | (oxygen saturation* or oxygen-h?emoglobin saturation or oximetries or oximetry or pulse oximet* or anox?emia*or anoxia or hypox?emia or hypoxia or oxygen deficienc* or (shock index* or shock indices)).mp. | 23891 |
| 13 | Body Temperature/ or Body Temperature Regulation/ or Skin Temperature/ or Hypothermia/ or Hypothermia, Induced/ | 4711 |
| 14 | (body temperature* or organ temperature* or heat loss* or thermoregulation* or skin temperature* or hypothermia*).mp. | 12725 |
| 15 | Reflex, Pupillary/ | 100 |
| 16 | (pupil* size or pupil* diameter* or pupil* reflex or pupil* light reflex or pupillometry or pupil* abnormalit* or pupil* examination* or pupil* measurement* or pupil* response or pupil* dilation or pupil* constriction or dilated pupils or (pupil* adj1 (react* or unreactiv*))).mp. | 2071 |
| 17 | Glasgow Coma Scale/ | 471 |
| 18 | (Glasgow coma scale* or Glasgow coma score* or GCS score or (AVPU or (Alert and Verbal and Pain and Unresponsive))).mp. | 2451 |
| 19 | Blood Alcohol Content/ | 40 |
| 20 | (blood alcohol adj (concentration* or content or level? or reading?)).mp. | 785 |
| 21 | Body Mass Index/ or Body Height/ or Body Weight/ | 18890 |
| 22 | (body height or body weight or obesity or overweight or underweight or (body height or body weight or obesity or overweight or underweight or body mass index)).mp. | 117080 |
| 23 | Fibrinogen/ | 1267 |
| 24 | (coagulation factor* or fibrinogen* or coagulation paramet*or fibrinolytic paramet* or blood coagulation or coagulation assay*).mp. | 9303 |
| 25 | Blood Platelets/ or exp Hemoglobins/ or h?emoglobin*.mp. | 45273 |
| 26 | International Normalized Ratio/ | 511 |
| 27 | (international normali?ed ratio* or INR level* or INR value* or (INR adj3 (admission or reversal))).mp. | 2897 |
| 28 | APACHE/ or Trauma Severity Indices/ or Abbreviated Injury Scale/ or Injury Severity Score/ | 1308 |
| 29 | (((trauma severity or abbreviated injury or injury severity or Abbreviated injury or Revised trauma or Trauma injury severity) adj (score* or scale* or index* or indices or measure*)) or ((ISS or AIS or RTS or TRISS) adj (scor* or scale* or index* or indices or measure*)) or (APACHE or "acute physiology and chronic health evaluation" or "acute physiolog* assessment and chronic health evaluation")).mp. | 4351 |
| 30 | (((injur* or trauma*) adj3 (characteristic* or nature or mechanism*)) or (associated adj (injur* or trauma*)) or ((concurrent or simultaneous* or accompanying) adj3 (injur* or trauma*))).mp. | 1755 |
| 31 | ((post-injury or postinjury or post-trauma* or posttrauma*) adj3 (complication* or infection*)).mp. | 131 |
| 32 | ((complication* or infection) adj3 (after or following) adj3 (injur* or trauma*)).mp. | 4480 |
| 33 | Coagulopath*.mp. | 1667 |
| 34 | ((time adj2 (transport or scene)) or (travel time* or definitive care or "time to emergency" or "time to hospital" or "time to transport*")).mp. | 2477 |
| 35 | (((delay* or optimal or optimum) adj3 (time* or timing)) or ((time* or timing) adj3 (intervention? or treatment*))).mp. | 87178 |
| 36 | (((intensive care or critical care or ICU or ITU or HDU) adj5 (day or days)) or ((ICU or ITU or HDU) adj (day or days or stay))).mp. | 7088 |
| 37 | "Length of Stay"/ | 7440 |
| 38 | ((length adj2 (hospital or stay)) or (prolonged adj (hospital* or ICU or ITU or HDU))).mp. | 29609 |
| 39 | "Transportation of Patients"/ or Ambulances/ or Air Ambulances/ or Patient Transfer/ | 421 |
| 40 | (((primary or secondary) adj (transport* or transfer*)) or (patient? adj1 (transport* or transfer*)) or (Interfacility or interhospital or Inter-facility or inter-hospital)).mp. | 2285 |
| 41 | (((post-injury or postinjury or post-trauma* or posttrauma*) adj3 amnes*) or (amnes* adj3 (after or following) adj3 (injur* or trauma*))).mp. | 128 |
| 42 | ((((mechanical* adj2 ventilat*) or (mechanical* adj2 respirat*) or (artificial* adj2 respirat*) or (artificial* adj2 ventilat*)) adj15 (length or prolonged or duration or time?)) or (ventilat* adj5 (day or days or hours))).mp. | 9065 |
| 43 | or/9-42 | 419359 |
| 44 | (prognostic index or prognostic indices or prognostic rule* or prognostic abilit* or prognostic sign* or prognostic data or (laboratory value* or clinical value* or predictive abilit* or predictive values) or (indicator* and (predict* or prognos*)) or (predict* adj8 prognos*) or (laboratory value* or clinical value* or predictive abilit* or predictive values) or ((scor* or algorithm* or model* or variable* or machine learning) adj3 (predict* or prognos*)) or ((rule* or finding* or criteria or validat* or observ* or paramet*) adj3 (predict* or prognos*)) or (model* adj9 variable*) or (risk? adj5 (predict* or prognos*)) or ((mortalit* or survival) adj2 (predict* or prognos* or overpredict* or underpredict*)) or ((mortalit* or survival or outcome*) adj9 (indicator* or determinant*)) or ((multivariab* or multivariat* or multi-variab* or multi-variat* or univariab* or univariat* or logistic regression model* or logistic regression analys*) adj5 (prognos* or predict* or outcome* or endpoint* or end-point*)) or ((predict* or prognos* or indicator*) adj3 (recovery or functional)) or (("area* under the curve" or "area under curve" or "area under receive" or "area under the receiver" or "receiver operat* characteristic") and (prognos* or predict*)) or ((validation or stratification or discrimination or calibration or indices) adj15 (prognos* or predict*)) or ((predict* or prognos*) adj1 outcome*) or (prognostication* or clinical indicator* or clinical variable* or clinical characteristic* or clinical factor*) or ((factor* or characteristic* or feature*) adj3 (prognos* or predict*))).mp. | 93728 |
| 45 | 8 and 43 and 44 | 147 |
| 46 | limit 45 to english language | 118 |

###### **CINAHL = 31,843**

| **#** | **Query** | **Results** |
| --- | --- | --- |
| S42 | S39 NOT S40  Limiters - English Language | 1,976 |
| S41 | S39 NOT S40 | 1,997 |
| S40 | S7 AND S37 AND S38  Limiters - Publication Type: Anecdote, Bibliography, Biography, Book, Book Chapter, Book Review, Brief Item, Care Plan, Case Study, CEU, Commentary, Consumer/Patient Teaching Materials, Directories, Doctoral Dissertation, Editorial, Exam Questions, Letter, Masters Thesis, Pamphlet, Proceedings, Review, Systematic Review | 241 |
| S39 | S7 AND S37 AND S38 | 2,238 |
| S38 | (“prognostic index” or “prognostic indices” or “prognostic rule*” or “prognostic abilit*” or “prognostic sign*” or “prognostic data” or (“laboratory value*” or “clinical value*” or “predictive abilit*” or “predictive values”) or (indicator* and (predict* or prognos*)) or (predict* N7 prognos*) or ((scor* or algorithm* or model* or variable* or machine learning) N2 (predict* or prognos*)) or ((rule* or finding* or criteria or validat* or observ* or paramet*) N2 (predict* or prognos*)) or (model* N8 variable*) or (risk? N4 (predict* or prognos*)) or ((mortalit* or survival) N1 (predict* or prognos* or overpredict* or underpredict*)) or ((mortalit* or survival or outcome*) N8 (indicator* or determinant*)) or ((multivariab* or multivariat* or multi-variab* or multi-variat* or univariab* or univariat* or “logistic regression model*” or “logistic regression analys*”) N4 (prognos* or predict* or outcome* or endpoint* or “end-point*”)) or ((predict* or prognos* or indicator*) N2 (recovery or functional)) or (("area* under the curve" or "area under curve" or "area under receive" or "area under the receiver" or "receiver operat* characteristic") and (prognos* or predict*)) or ((validation or stratification or discrimination or calibration or indices) N14 (prognos* or predict*)) or ((predict* or prognos*) N1 outcome*) or (prognostication* or “clinical indicator*” or “clinical variable*” or “clinical characteristic*” or “clinical factor*”) or ((factor* or characteristic* or feature*) N2 (prognos* or predict*))) | 286,971 |
| S37 | S8 OR S9 OR S10 OR S11 OR S12 OR S13 OR S14 OR S15 OR S16 OR S17 OR S18 OR S19 OR S20 OR S21 OR S22 OR S23 OR S24 OR S25 OR S26 OR S27 OR S28 OR S29 OR S30 OR S31 OR S32 OR S33 OR S34 OR S35 OR S36 | 604,065 |
| S36 | ( (((mechanical* N1 ventilat*) or (mechanical* N1 respirat*) or (artificial* N1 respirat*) or (artificial* N1 ventilat*)) N14 (length or prolonged or duration or time?)) ) OR ( (ventilat* N4 (day or days or hours))) ) | 8,590 |
| S35 | ( (("post-injury" or postinjury or "post-trauma*" or posttrauma*) N2 amnes*) ) OR ( (amnes* N2 (after or following) N2 (injur* or trauma*)) ) | 432 |
| S34 | ( ((primary or secondary) N0 (transport* or transfer*)) ) OR ( (patient? N1 (transport* or transfer*)) ) OR ( (Interfacility or interhospital or "Inter-facility" or "inter-hospital") ) | 12,387 |
| S33 | ( (length N1 (hospital or stay)) ) OR ( (prolonged N0 (hospital* or ICU or ITU or HDU)) ) | 66,897 |
| S32 | ( (("intensive care" or "critical care" or ICU or ITU or HDU) N4 (day or days)) ) OR ( ((ICU or ITU or HDU) N0 (day or days or stay)) ) | 7,373 |
| S31 | ( ((delay* or optimal or optimum) N2 (time* or timing)) ) OR ( ((time* or timing) N2 (intervention? or treatment*)) ) | 31,597 |
| S30 | ( (time N1 (transport or scene)) ) OR ( ("travel time*" or "definitive care" or "time to emergency" or "time to hospital" or "time to transport*")) ) | 2,353 |
| S29 | Coagulopath* | 3,899 |
| S28 | ((complication* or infection) N2 (after or following) N2 (injur* or trauma*)) | 608 |
| S27 | (("post-injury" or postinjury or "post-trauma*" or posttrauma*) N2 (complication* or infection*)) | 1,534 |
| S26 | ( ((injur* or trauma*) N2 (characteristic* or nature or mechanism*)) ) OR ( (associated N0 (injur* or trauma*)) ) OR ( ((concurrent or simultaneous* or accompanying) N2 (injur* or trauma*)) ) | 15,249 |
| S25 | ( (("trauma severity" or "injury severity" or "Abbreviated injury" or "Revised trauma" or "Trauma injury severity") N0 (score* or scale* or index* or indices or measure*)) ) OR ( ((ISS or AIS or RTS or TRISS) N0 (scor* or scale* or index* or indices or measure*)) ) OR ( (APACHE or "acute physiology and chronic health evaluation" or "acute physiolog* assessment and chronic health evaluation")) ) | 19,772 |
| S24 | (MH "Apache") OR (MH "Trauma Severity Indices") | 15,805 |
| S23 | ( ("international normali?ed ratio*" or "INR level*" or "INR value*") ) OR ( (INR N2 (admission or reversal)) ) | 4,728 |
| S22 | ("Blood Platelet*" or h?emoglobin*) | 13,756 |
| S21 | ("coagulation factor*" or fibrinogen* or "coagulation paramet*" or "fibrinolytic paramet*" or "blood coagulation" or "coagulation assay*") | 20,426 |
| S20 | ("body height" or "body weight" or obesity or overweight or underweight or "body mass index") | 267,301 |
| S19 | (MH "Body Mass Index") OR (MH "Body Height") OR (MH "Body Weight") | 118,551 |
| S18 | ("blood alcohol" N0 (concentration* or content or level? or reading?)) | 1,232 |
| S17 | ("Glasgow coma scale*" or "Glasgow coma score*" or "GCS score" or AVPU or "Alert and Verbal and Pain and Unresponsive") | 10,046 |
| S16 | (MH "Glasgow Coma Scale") | 8,692 |
| S15 | ( ("pupil* size" or "pupil* diameter*" or "pupil* reflex" or "pupil* light reflex" or pupillometry or "pupil* abnormalit*" or "pupil* examination*" or "pupil* measurement*" or "pupil* response" or "pupil* dilation" or "pupil* constriction" or "dilated pupils") ) OR ( (pupil* N1 (react* or unreactiv*)) ) | 1,695 |
| S14 | (MH "Reflex, Pupillary") | 454 |
| S13 | ("body temperature*" or "organ temperature*" or "heat loss*" or thermoregulation* or "skin temperature*" or hypothermia*) | 22,287 |
| S12 | (MH "Body Temperature Regulation") OR (MH "Body Temperature") OR (MH "Core Body Temperature") OR (MH "Skin Temperature") OR (MH "Hypothermia") OR (MH "Hypothermia, Induced") | 16,402 |
| S11 | ("oxygen saturation*" or "oxygen-h?emoglobin saturation" or oximetries or oximetry or "pulse oximet*" or anox?emia*or anoxia or hypox?emia or hypoxia or "oxygen deficienc*" or "shock index*" or "shock indices") | 30,485 |
| S10 | (MH "Oximetry") OR (MH "Pulse Oximetry") | 5,368 |
| S9 | ("physiological observation?" or "clinical observation?" or "cardiac rate?" or "heart rate?" or heartbeat? or "pulse rate?" or "vital sign?" or "blood pressure" or "diastolic pressure" or "pulse pressure" or "systolic pressure") | 143,646 |
| S8 | (MH "Vital Signs") OR (MH "Blood Pressure Determination") OR (MH "Body Temperature Determination") OR (MH "Pulse") | 17,581 |
| S7 | S1 OR S2 OR S3 OR S4 OR S5 OR S6 | 58,323 |
| S6 | ("traumatic lesion*" N2 (Brain* or Cerebr* or Cerebel* or Cortex or Crani* or "Diffuse Axonal" or Head or Hemisphere* or "Frontal Lobe*" or "Temporal Lobe*" or "Occipital Lobe*" or "Parietal Lobe*" or intracranial* or encephalopath* or bulbar or medullary or midbrain or pontine or subarachnoid or subdural)) | 9 |
| S5 | ( ((severe or moderate or mild) N0 GCS) ) OR ( ("GCS score" N2 ("3" or "4" or "5" or "6" or "7" or "8" or "9" or "10" or "11" or "12")) ) OR ( (("glasgow coma scale*" or "glasgow outcome scale" or "glasgow scale*" or "Rancho Los Amigos") N0 ("3" or "4" or "5" or "6" or "7" or "8" or "9" or "10" or "11" or "12")) ) | 455 |
| S4 | ( (penetrat* N2 skull* N2 (trauma* or injur* or fracture*)) ) OR ( (penetrat* N2 head* N2 (wound* or fracture*)) ) | 23 |
| S3 | ( (Concuss* or Postconcuss* or Pneumocephalus or "Crush* Skull*" or "brain commotion?" or "Commotio Cerebri" or "Contusio Cerebri" or "posttraumatic coma*" or "post-traumatic coma*" or "depressed skull fracture*") ) OR ( ((Brain* or Cerebr* or Cerebel* or "Diffuse Axonal" or intracrani*) N0 (damage* or wound* or lacerat* or contusion?)) ) | 13,620 |
| S2 | ( ((Brain* or Cerebr* or Cerebel* or Cortex or Crani* or "Diffuse Axonal" or Head or Hemisphere* or "Frontal Lobe*" or "Temporal Lobe*" or "Occipital Lobe*" or "Parietal Lobe*" or intracranial*) N1 (Injur* or Trauma*)) ) OR ( ((Posttraumatic or Traumatic) N0 (brain* or encephalopath* or intracrani* or cerebel* or cerebr* or craniocerebr*)) ) OR ( ((traumatic or posttraumatic) N0 (brainstem or bulbar or medullary or midbrain or pontine or subarachnoid or subdural) N0 (h?em?or*ag* or h?ematoma*)) ) | 49,527 |
| S1 | (MH "Brain Injuries") OR (MH "Brain Concussion") OR (MH "Postconcussion Syndrome") OR (MH "Brain Contusions") OR (MH "Chronic Traumatic Encephalopathy") OR (MH "Pneumocephalus") OR (MH "Left Hemisphere Injuries") OR (MH "Right Hemisphere Injuries") | 31,843 |

###### **EMBASE = 4,564**

Database(s): **Embase Classic+Embase**1947 to 2022 February 25
Search Strategy:

| **#** | **Searches** | **Results** |
| --- | --- | --- |
| 1 | brain injury/ or acquired brain injury/ or brain concussion/ or brain contusion/ or brain damage/ or brain stem injury/ or cerebellum injury/ or diffuse brain injury/ or postconcussion syndrome/ or traumatic brain injury/ or diffuse axonal injury/ or traumatic brain injury/ or chronic traumatic encephalopathy/ or pediatric traumatic brain injury/ or head injury/ or depressed skull fracture/ or pneumocephalus/ | 254689 |
| 2 | ((Brain* or Cerebr* or Cerebel* or Cortex or Crani* or Diffuse Axonal or Head or Hemisphere* or Frontal Lobe* or Temporal Lobe* or Occipital Lobe* or Parietal Lobe* or intracranial*) adj1 (Injur* or Trauma*)).mp,kf,kw,hw. | 246222 |
| 3 | (((Posttraumatic or Traumatic) adj (brain* or encephalopath* or intracrani* or cerebel* or cerebr* or craniocerebr*)) or ((traumatic or posttraumatic) adj (brainstem or bulbar or medullary or midbrain or pontine or subarachnoid or subdural) adj (h?em?or*ag* or h?ematoma*))).mp,kf,kw,hw. | 81026 |
| 4 | (Concuss* or Postconcuss* or Pneumocephalus or Crush* Skull* or brain commotion? or Commotio Cerebri or Contusio Cerebri or posttraumatic coma* or post-traumatic coma* or depressed skull fracture* or ((Brain* or Cerebr* or Cerebel* or Diffuse Axonal or intracrani*) adj (damage* or wound* or lacerat* or contusion?))).mp,kf,kw,hw. | 99191 |
| 5 | ((penetrat* adj3 skull* adj3 (trauma* or injur* or fracture*)) or (penetrat* adj3 head* adj3 (wound* or fracture*))).mp,kf,kw,hw. | 173 |
| 6 | (((severe or moderate or mild) adj GCS) or (GCS score adj3 ("3" or "4" or "5" or "6" or "7" or "8" or "9" or "10" or "11" or "12")) or ((glasgow coma scale* or glasgow outcome scale or glasgow scale* or Rancho Los Amigos) adj ("3" or "4" or "5" or "6" or "7" or "8" or "9" or "10" or "11" or "12"))).mp,kf,kw,hw. | 2072 |
| 7 | (traumatic lesion* adj3 (Brain* or Cerebr* or Cerebel* or Cortex or Crani* or Diffuse Axonal or Head or Hemisphere* or Frontal Lobe* or Temporal Lobe* or Occipital Lobe* or Parietal Lobe* or intracranial* or encephalopath* or bulbar or medullary or midbrain or pontine or subarachnoid or subdural)).mp,kf,kw,hw. | 218 |
| 8 | 1 or 2 or 3 or 4 or 5 or 6 or 7 | 314584 |
| 9 | vital signs/ or blood pressure/ or heart rate/ | 518021 |
| 10 | (cardiac rate? or heart rate? or heartbeat? or pulse rate? or vital sign? or (physiological observation? or clinical observation?)).mp. | 505454 |
| 11 | (blood pressure or diastolic pressure or pulse pressure or systolic pressure).mp. | 743329 |
| 12 | oximetry/ or hypoxia/ | 137076 |
| 13 | (oxygen saturation* or oxygen-h?emoglobin saturation or oximetries or oximetry or pulse oximet* or anox?emia*or anoxia or hypox?emia or hypoxia or oxygen deficienc* or (shock index* or shock indices)).mp. | 389680 |
| 14 | Body Temperature/ or Body Temperature Regulation/ or Skin Temperature/ or Hypothermia/ or Hypothermia, Induced/ | 148034 |
| 15 | (body temperature* or organ temperature* or heat loss* or thermoregulation* or skin temperature* or hypothermia*).mp. | 183591 |
| 16 | Pupillary Reflex/ | 6291 |
| 17 | (pupil* size or pupil* diameter* or pupil* reflex or pupil* light reflex or pupillometry or pupil* abnormalit* or pupil* examination* or pupil* measurement* or pupil* response or pupil* dilation or pupil* constriction or dilated pupils or (pupil* adj1 (react* or unreactiv*))).mp. | 19124 |
| 18 | Glasgow Coma Scale/ | 33189 |
| 19 | (Glasgow coma scale* or Glasgow coma score* or GCS score or (AVPU or (Alert and Verbal and Pain and Unresponsive))).mp. | 38248 |
| 20 | Blood Alcohol Content/ | 11156 |
| 21 | (blood alcohol adj (concentration* or content or level? or reading?)).mp. | 6640 |
| 22 | Body Mass Index/ or Body Height/ or Body Weight/ | 811948 |
| 23 | (body height or body weight or obesity or overweight or underweight or (body height or body weight or obesity or overweight or underweight or body mass index)).mp. | 1340717 |
| 24 | Fibrinogen/ | 79281 |
| 25 | (coagulation factor* or fibrinogen* or coagulation paramet*or fibrinolytic paramet* or blood coagulation or coagulation assay*).mp. | 155805 |
| 26 | Blood Platelets/ or exp Hemoglobins/ or h?emoglobin*.mp. | 611008 |
| 27 | International Normalized Ratio/ | 41470 |
| 28 | (international normali?ed ratio* or INR level* or INR value* or (INR adj3 (admission or reversal))).mp. | 45197 |
| 29 | APACHE/ or Trauma Severity Indices/ or Abbreviated Injury Scale/ or Injury Severity Score/ | 44900 |
| 30 | (((trauma severity or abbreviated injury or injury severity or Abbreviated injury or Revised trauma or Trauma injury severity) adj (score* or scale* or index* or indices or measure*)) or ((ISS or AIS or RTS or TRISS) adj (scor* or scale* or index* or indices or measure*)) or (APACHE or "acute physiology and chronic health evaluation" or "acute physiolog* assessment and chronic health evaluation")).mp. | 43990 |
| 31 | (((injur* or trauma*) adj3 (characteristic* or nature or mechanism*)) or (associated adj (injur* or trauma*)) or ((concurrent or simultaneous* or accompanying) adj3 (injur* or trauma*))).mp. | 54734 |
| 32 | ((post-injury or postinjury or post-trauma* or posttrauma*) adj3 (complication* or infection*)).mp. | 2568 |
| 33 | ((complication* or infection) adj3 (after or following) adj3 (injur* or trauma*)).mp. | 3075 |
| 34 | Coagulopath*.mp. | 29538 |
| 35 | ((time adj2 (transport or scene)) or (travel time* or definitive care or "time to emergency" or "time to hospital" or "time to transport*") or (((delay* or optimal or optimum) adj3 (time* or timing)) or ((time* or timing) adj3 (intervention? or treatment*)))).mp. | 245910 |
| 36 | (((intensive care or critical care or ICU or ITU or HDU) adj5 (day or days)) or ((ICU or ITU or HDU) adj (day or days or stay))).mp. | 42425 |
| 37 | "Length of Stay"/ | 229642 |
| 38 | ((length adj2 (hospital or stay)) or (prolonged adj (hospital* or ICU or ITU or HDU))).mp. | 279441 |
| 39 | "Transportation of Patients"/ or Ambulances/ or Air Ambulances/ or Patient Transfer/ | 45019 |
| 40 | (((primary or secondary) adj (transport* or transfer*)) or (patient? adj1 (transport* or transfer*)) or (Interfacility or interhospital or Inter-facility or inter-hospital)).mp. | 43591 |
| 41 | (((post-injury or postinjury or post-trauma* or posttrauma*) adj3 amnes*) or (amnes* adj3 (after or following) adj3 (injur* or trauma*))).mp. | 1408 |
| 42 | ((((mechanical* adj2 ventilat*) or (mechanical* adj2 respirat*) or (artificial* adj2 respirat*) or (artificial* adj2 ventilat*)) adj15 (length or prolonged or duration or time?)) or (ventilat* adj5 (day or days or hours))).mp. | 42028 |
| 43 | or/9-42 | 4115388 |
| 44 | (prognostic index or prognostic indices or prognostic rule* or prognostic abilit* or prognostic sign* or prognostic data or (laboratory value* or clinical value* or predictive abilit* or predictive values) or (indicator* and (predict* or prognos*)) or (predict* adj8 prognos*) or (laboratory value* or clinical value* or predictive abilit* or predictive values) or ((scor* or algorithm* or model* or variable* or machine learning) adj3 (predict* or prognos*)) or ((rule* or finding* or criteria or validat* or observ* or paramet*) adj3 (predict* or prognos*)) or (model* adj9 variable*) or (risk? adj5 (predict* or prognos*)) or ((mortalit* or survival) adj2 (predict* or prognos* or overpredict* or underpredict*)) or ((mortalit* or survival or outcome*) adj9 (indicator* or determinant*)) or ((multivariab* or multivariat* or multi-variab* or multi-variat* or univariab* or univariat* or logistic regression model* or logistic regression analys*) adj5 (prognos* or predict* or outcome* or endpoint* or end-point*)) or ((predict* or prognos* or indicator*) adj3 (recovery or functional)) or (("area* under the curve" or "area under curve" or "area under receive" or "area under the receiver" or "receiver operat* characteristic") and (prognos* or predict*)) or ((validation or stratification or discrimination or calibration or indices) adj15 (prognos* or predict*)) or ((predict* or prognos*) adj1 outcome*) or (prognostication* or clinical indicator* or clinical variable* or clinical characteristic* or clinical factor*) or ((factor* or characteristic* or feature*) adj3 (prognos* or predict*))).mp. | 1625012 |
| 45 | 8 and 43 and 44 | 9232 |
| 46 | limit 45 to english language | 8784 |
| 47 | (exp animal/ or exp invertebrate/ or animal.hw. or nonhuman/) not exp human/ | 7676908 |
| 48 | 46 not 47 | 8571 |
| 49 | limit 48 to (conference abstract or editorial or erratum or letter or note) | 2237 |
| 50 | 48 not 49 | 6334 |
| 51 | limit 50 to "review" | 615 |
| 52 | 50 not 51 | 5719 |
| 53 | 52 not (address or bibliograph* or case report* or conference or comment* or congress* or editorial* or letter* or news or newspaper?).mp. | 5294 |
| 54 | 53 not (Anecdote* or Biograph* or Book or Book Chapter* or Book Review* or, Brief Item* or Case Study or Directories or Dissertation or Thesis or Theses or Exam Questions or Pamphlet or Proceedings).af. | 5238 |
| 55 | limit 54 to medline | 674 |
| 56 | 54 not 55 | 4564 |

###### **EMCARE= 2,253**

Database(s): **Ovid Emcare**1995 to 2022 Week 8
Search Strategy:

| **#** | **Searches** | **Results** |
| --- | --- | --- |
| 1 | brain injury/ or acquired brain injury/ or brain concussion/ or brain contusion/ or brain damage/ or brain stem injury/ or cerebellum injury/ or diffuse brain injury/ or postconcussion syndrome/ or traumatic brain injury/ or diffuse axonal injury/ or traumatic brain injury/ or chronic traumatic encephalopathy/ or pediatric traumatic brain injury/ or head injury/ or depressed skull fracture/ or pneumocephalus/ | 63979 |
| 2 | ((Brain* or Cerebr* or Cerebel* or Cortex or Crani* or Diffuse Axonal or Head or Hemisphere* or Frontal Lobe* or Temporal Lobe* or Occipital Lobe* or Parietal Lobe* or intracranial*) adj1 (Injur* or Trauma*)).mp,kf,kw,hw. | 61971 |
| 3 | (((Posttraumatic or Traumatic) adj (brain* or encephalopath* or intracrani* or cerebel* or cerebr* or craniocerebr*)) or ((traumatic or posttraumatic) adj (brainstem or bulbar or medullary or midbrain or pontine or subarachnoid or subdural) adj (h?em?or*ag* or h?ematoma*))).mp,kf,kw,hw. | 25425 |
| 4 | (Concuss* or Postconcuss* or Pneumocephalus or Crush* Skull* or brain commotion? or Commotio Cerebri or Contusio Cerebri or posttraumatic coma* or post-traumatic coma* or depressed skull fracture* or ((Brain* or Cerebr* or Cerebel* or Diffuse Axonal or intracrani*) adj (damage* or wound* or lacerat* or contusion?))).mp,kf,kw,hw. | 21839 |
| 5 | ((penetrat* adj3 skull* adj3 (trauma* or injur* or fracture*)) or (penetrat* adj3 head* adj3 (wound* or fracture*))).mp,kf,kw,hw. | 23 |
| 6 | (((severe or moderate or mild) adj GCS) or (GCS score adj3 ("3" or "4" or "5" or "6" or "7" or "8" or "9" or "10" or "11" or "12")) or ((glasgow coma scale* or glasgow outcome scale or glasgow scale* or Rancho Los Amigos) adj ("3" or "4" or "5" or "6" or "7" or "8" or "9" or "10" or "11" or "12"))).mp,kf,kw,hw. | 580 |
| 7 | (traumatic lesion* adj3 (Brain* or Cerebr* or Cerebel* or Cortex or Crani* or Diffuse Axonal or Head or Hemisphere* or Frontal Lobe* or Temporal Lobe* or Occipital Lobe* or Parietal Lobe* or intracranial* or encephalopath* or bulbar or medullary or midbrain or pontine or subarachnoid or subdural)).mp,kf,kw,hw. | 22 |
| 8 | 1 or 2 or 3 or 4 or 5 or 6 or 7 | 76046 |
| 9 | vital signs/ or blood pressure/ or heart rate/ | 103709 |
| 10 | (cardiac rate? or heart rate? or heartbeat? or pulse rate? or vital sign? or (physiological observation? or clinical observation?)).mp. | 112788 |
| 11 | (blood pressure or diastolic pressure or pulse pressure or systolic pressure).mp. | 140860 |
| 12 | oximetry/ or hypoxia/ | 20715 |
| 13 | (oxygen saturation* or oxygen-h?emoglobin saturation or oximetries or oximetry or pulse oximet* or anox?emia*or anoxia or hypox?emia or hypoxia or oxygen deficienc* or (shock index* or shock indices)).mp. | 75652 |
| 14 | Body Temperature/ or Body Temperature Regulation/ or Skin Temperature/ or Hypothermia/ or Hypothermia, Induced/ | 28640 |
| 15 | (body temperature* or organ temperature* or heat loss* or thermoregulation* or skin temperature* or hypothermia*).mp. | 33156 |
| 16 | Pupillary Reflex/ | 1280 |
| 17 | (pupil* size or pupil* diameter* or pupil* reflex or pupil* light reflex or pupillometry or pupil* abnormalit* or pupil* examination* or pupil* measurement* or pupil* response or pupil* dilation or pupil* constriction or dilated pupils or (pupil* adj1 (react* or unreactiv*))).mp. | 3487 |
| 18 | Glasgow Coma Scale/ | 11920 |
| 19 | (Glasgow coma scale* or Glasgow coma score* or GCS score or (AVPU or (Alert and Verbal and Pain and Unresponsive))).mp. | 12896 |
| 20 | Blood Alcohol Content/ | 3922 |
| 21 | (blood alcohol adj (concentration* or content or level? or reading?)).mp. | 1984 |
| 22 | Body Mass Index/ or Body Height/ or Body Weight/ | 219473 |
| 23 | (body height or body weight or obesity or overweight or underweight or (body height or body weight or obesity or overweight or underweight or body mass index)).mp. | 321540 |
| 24 | Fibrinogen/ | 10599 |
| 25 | (coagulation factor* or fibrinogen* or coagulation paramet*or fibrinolytic paramet* or blood coagulation or coagulation assay*).mp. | 19721 |
| 26 | Blood Platelets/ or exp Hemoglobins/ or h?emoglobin*.mp. | 108555 |
| 27 | International Normalized Ratio/ | 9490 |
| 28 | (international normali?ed ratio* or INR level* or INR value* or (INR adj3 (admission or reversal))).mp. | 10080 |
| 29 | APACHE/ or Trauma Severity Indices/ or Abbreviated Injury Scale/ or Injury Severity Score/ | 15347 |
| 30 | (((trauma severity or abbreviated injury or injury severity or Abbreviated injury or Revised trauma or Trauma injury severity) adj (score* or scale* or index* or indices or measure*)) or ((ISS or AIS or RTS or TRISS) adj (scor* or scale* or index* or indices or measure*)) or (APACHE or "acute physiology and chronic health evaluation" or "acute physiolog* assessment and chronic health evaluation")).mp. | 14768 |
| 31 | (((injur* or trauma*) adj3 (characteristic* or nature or mechanism*)) or (associated adj (injur* or trauma*)) or ((concurrent or simultaneous* or accompanying) adj3 (injur* or trauma*))).mp. | 18055 |
| 32 | ((post-injury or postinjury or post-trauma* or posttrauma*) adj3 (complication* or infection*)).mp. | 984 |
| 33 | ((complication* or infection) adj3 (after or following) adj3 (injur* or trauma*)).mp. | 811 |
| 34 | Coagulopath*.mp. | 6039 |
| 35 | ((time adj2 (transport or scene)) or (travel time* or definitive care or "time to emergency" or "time to hospital" or "time to transport*") or (((delay* or optimal or optimum) adj3 (time* or timing)) or ((time* or timing) adj3 (intervention? or treatment*)))).mp. | 55688 |
| 36 | (((intensive care or critical care or ICU or ITU or HDU) adj5 (day or days)) or ((ICU or ITU or HDU) adj (day or days or stay))).mp. | 10617 |
| 37 | "Length of Stay"/ | 67890 |
| 38 | ((length adj2 (hospital or stay)) or (prolonged adj (hospital* or ICU or ITU or HDU))).mp. | 79181 |
| 39 | "Transportation of Patients"/ or Ambulances/ or Air Ambulances/ or Patient Transfer/ | 19077 |
| 40 | (((primary or secondary) adj (transport* or transfer*)) or (patient? adj1 (transport* or transfer*)) or (Interfacility or interhospital or Inter-facility or inter-hospital)).mp. | 16230 |
| 41 | (((post-injury or postinjury or post-trauma* or posttrauma*) adj3 amnes*) or (amnes* adj3 (after or following) adj3 (injur* or trauma*))).mp. | 490 |
| 42 | ((((mechanical* adj2 ventilat*) or (mechanical* adj2 respirat*) or (artificial* adj2 respirat*) or (artificial* adj2 ventilat*)) adj15 (length or prolonged or duration or time?)) or (ventilat* adj5 (day or days or hours))).mp. | 11564 |
| 43 | or/9-42 | 894282 |
| 44 | (prognostic index or prognostic indices or prognostic rule* or prognostic abilit* or prognostic sign* or prognostic data or (laboratory value* or clinical value* or predictive abilit* or predictive values) or (indicator* and (predict* or prognos*)) or (predict* adj8 prognos*) or (laboratory value* or clinical value* or predictive abilit* or predictive values) or ((scor* or algorithm* or model* or variable* or machine learning) adj3 (predict* or prognos*)) or ((rule* or finding* or criteria or validat* or observ* or paramet*) adj3 (predict* or prognos*)) or (model* adj9 variable*) or (risk? adj5 (predict* or prognos*)) or ((mortalit* or survival) adj2 (predict* or prognos* or overpredict* or underpredict*)) or ((mortalit* or survival or outcome*) adj9 (indicator* or determinant*)) or ((multivariab* or multivariat* or multi-variab* or multi-variat* or univariab* or univariat* or logistic regression model* or logistic regression analys*) adj5 (prognos* or predict* or outcome* or endpoint* or end-point*)) or ((predict* or prognos* or indicator*) adj3 (recovery or functional)) or (("area* under the curve" or "area under curve" or "area under receive" or "area under the receiver" or "receiver operat* characteristic") and (prognos* or predict*)) or ((validation or stratification or discrimination or calibration or indices) adj15 (prognos* or predict*)) or ((predict* or prognos*) adj1 outcome*) or (prognostication* or clinical indicator* or clinical variable* or clinical characteristic* or clinical factor*) or ((factor* or characteristic* or feature*) adj3 (prognos* or predict*))).mp. | 372531 |
| 45 | 8 and 43 and 44 | 2961 |
| 46 | limit 45 to english language | 2875 |
| 47 | (exp animal/ or exp invertebrate/ or animal.hw. or nonhuman/) not exp human/ | 494670 |
| 48 | 46 not 47 | 2842 |
| 49 | limit 48 to (conference abstract or editorial or erratum or letter or note) | 66 |
| 50 | 48 not 49 | 2776 |
| 51 | limit 50 to "review" | 276 |
| 52 | 50 not 51 | 2500 |
| 53 | 52 not (address or bibliograph* or case report* or conference or comment* or congress* or editorial* or letter* or news or newspaper?).mp. | 2272 |
| 54 | 53 not (Anecdote* or Biograph* or Book or Book Chapter* or Book Review* or, Brief Item* or Case Study or Directories or Dissertation or Thesis or Theses or Exam Questions or Pamphlet or Proceedings).af. | 2253 |

######

###### **MEDLINE= 4,805**

Database(s): **Ovid MEDLINE(R) and Epub Ahead of Print, In-Process, In-Data-Review & Other Non-Indexed Citations, Daily and Versions**1946 to February 25, 2022
Search Strategy:

| **#** | **Searches** | **Results** |
| --- | --- | --- |
| 1 | craniocerebral trauma/ or brain injuries/ or brain hemorrhage, traumatic/ or brain stem hemorrhage, traumatic/ or cerebral hemorrhage, traumatic/ or brain injuries, diffuse/ or diffuse axonal injury/ or brain injuries, traumatic/ or brain concussion/ or brain contusion/ or chronic traumatic encephalopathy/ or brain injury, chronic/ or pneumocephalus/ or coma, post-head injury/ or head injuries, closed/ or contrecoup injury/ or post-concussion syndrome/ or head injuries, penetrating/ or intracranial hemorrhage, traumatic/ or subarachnoid hemorrhage, traumatic/ or skull fracture, depressed/ | 99026 |
| 2 | ((Brain* or Cerebr* or Cerebel* or Cortex or Crani* or Diffuse Axonal or Head or Hemisphere* or Frontal Lobe* or Temporal Lobe* or Occipital Lobe* or Parietal Lobe* or intracranial*) adj1 (Injur* or Trauma*)).mp,kf,kw,hw. | 145675 |
| 3 | (((Posttraumatic or Traumatic) adj (brain* or encephalopath* or intracrani* or cerebel* or cerebr* or craniocerebr*)) or ((traumatic or posttraumatic) adj (brainstem or bulbar or medullary or midbrain or pontine or subarachnoid or subdural) adj (h?em?or*ag* or h?ematoma*))).mp,kf,kw,hw. | 47195 |
| 4 | (Concuss* or Postconcuss* or Pneumocephalus or Crush* Skull* or brain commotion? or Commotio Cerebri or Contusio Cerebri or posttraumatic coma* or post-traumatic coma* or depressed skull fracture* or ((Brain* or Cerebr* or Cerebel* or Diffuse Axonal or intracrani*) adj (damage* or wound* or lacerat* or contusion?))).mp,kf,kw,hw. | 58006 |
| 5 | ((penetrat* adj3 skull* adj3 (trauma* or injur* or fracture*)) or (penetrat* adj3 head* adj3 (wound* or fracture*))).mp,kf,kw,hw. | 117 |
| 6 | (((severe or moderate or mild) adj GCS) or (GCS score adj3 ("3" or "4" or "5" or "6" or "7" or "8" or "9" or "10" or "11" or "12")) or ((glasgow coma scale* or glasgow outcome scale or glasgow scale* or Rancho Los Amigos) adj ("3" or "4" or "5" or "6" or "7" or "8" or "9" or "10" or "11" or "12"))).mp,kf,kw,hw. | 1358 |
| 7 | (traumatic lesion* adj3 (Brain* or Cerebr* or Cerebel* or Cortex or Crani* or Diffuse Axonal or Head or Hemisphere* or Frontal Lobe* or Temporal Lobe* or Occipital Lobe* or Parietal Lobe* or intracranial* or encephalopath* or bulbar or medullary or midbrain or pontine or subarachnoid or subdural)).mp,kf,kw,hw. | 125 |
| 8 | 1 or 2 or 3 or 4 or 5 or 6 or 7 | 183244 |
| 9 | vital signs/ or blood pressure/ or heart rate/ | 385879 |
| 10 | (physiological observation? or clinical observation? or (cardiac rate? or heart rate? or heartbeat? or pulse rate? or vital sign?) or (blood pressure or diastolic pressure or pulse pressure or systolic pressure)).mp. | 673433 |
| 11 | oximetry/ or hypoxia/ | 81019 |
| 12 | (oxygen saturation* or oxygen-h?emoglobin saturation or oximetries or oximetry or pulse oximet* or anox?emia*or anoxia or hypox?emia or hypoxia or oxygen deficienc* or (shock index* or shock indices)).mp. | 224058 |
| 13 | Body Temperature/ or Body Temperature Regulation/ or Skin Temperature/ or Hypothermia/ or Hypothermia, Induced/ | 101233 |
| 14 | (body temperature* or organ temperature* or heat loss* or thermoregulation* or skin temperature* or hypothermia*).mp. | 134485 |
| 15 | Reflex, Pupillary/ | 2595 |
| 16 | (pupil* size or pupil* diameter* or pupil* reflex or pupil* light reflex or pupillometry or pupil* abnormalit* or pupil* examination* or pupil* measurement* or pupil* response or pupil* dilation or pupil* constriction or dilated pupils or (pupil* adj1 (react* or unreactiv*))).mp. | 11214 |
| 17 | Glasgow Coma Scale/ | 10257 |
| 18 | (Glasgow coma scale* or Glasgow coma score* or GCS score or (AVPU or (Alert and Verbal and Pain and Unresponsive))).mp. | 18487 |
| 19 | Blood Alcohol Content/ | 596 |
| 20 | (blood alcohol adj (concentration* or content or level? or reading?)).mp. | 4854 |
| 21 | Body Mass Index/ or Body Height/ or Body Weight/ | 337959 |
| 22 | (body height or body weight or obesity or overweight or underweight or (body height or body weight or obesity or overweight or underweight or body mass index)).mp. | 867208 |
| 23 | Fibrinogen/ | 33650 |
| 24 | (coagulation factor* or fibrinogen* or coagulation paramet* or fibrinolytic paramet* or blood coagulation or coagulation assay*).mp. | 150227 |
| 25 | Blood Platelets/ or exp Hemoglobins/ or h?emoglobin*.mp. | 323050 |
| 26 | International Normalized Ratio/ | 5918 |
| 27 | (international normali?ed ratio* or INR level* or INR value* or (INR adj3 (admission or reversal))).mp. | 12409 |
| 28 | APACHE/ or Trauma Severity Indices/ or Abbreviated Injury Scale/ or Injury Severity Score/ | 33329 |
| 29 | (((trauma severity or abbreviated injury or injury severity or Abbreviated injury or Revised trauma or Trauma injury severity) adj (score* or scale* or index* or indices or measure*)) or ((ISS or AIS or RTS or TRISS) adj (scor* or scale* or index* or indices or measure*)) or (APACHE or "acute physiology and chronic health evaluation" or "acute physiolog* assessment and chronic health evaluation")).mp. | 46447 |
| 30 | (((injur* or trauma*) adj3 (characteristic* or nature or mechanism*)) or (associated adj (injur* or trauma*)) or ((concurrent or simultaneous* or accompanying) adj3 (injur* or trauma*))).mp. | 41815 |
| 31 | ((post-injury or postinjury or post-trauma* or posttrauma*) adj3 (complication* or infection*)).mp. | 950 |
| 32 | ((complication* or infection) adj3 (after or following) adj3 (injur* or trauma*)).mp. | 2298 |
| 33 | Coagulopath*.mp. | 17780 |
| 34 | ((time adj2 (transport or scene)) or (travel time* or definitive care or "time to emergency" or "time to hospital" or "time to transport*")).mp. | 13657 |
| 35 | (((delay* or optimal or optimum) adj3 (time* or timing)) or ((time* or timing) adj3 (intervention? or treatment*))).mp. | 142343 |
| 36 | (((intensive care or critical care or ICU or ITU or HDU) adj5 (day or days)) or ((ICU or ITU or HDU) adj (day or days or stay))).mp. | 20583 |
| 37 | "Length of Stay"/ | 98363 |
| 38 | ((length adj2 (hospital or stay)) or (prolonged adj (hospital* or ICU or ITU or HDU))).mp. | 156492 |
| 39 | "Transportation of Patients"/ or Ambulances/ or Air Ambulances/ or Patient Transfer/ | 25934 |
| 40 | (((primary or secondary) adj (transport* or transfer*)) or (patient? adj1 (transport* or transfer*)) or (Interfacility or interhospital or Inter-facility or inter-hospital)).mp. | 18187 |
| 41 | (((post-injury or postinjury or post-trauma* or posttrauma*) adj3 amnes*) or (amnes* adj3 (after or following) adj3 (injur* or trauma*))).mp. | 924 |
| 42 | ((((mechanical* adj2 ventilat*) or (mechanical* adj2 respirat*) or (artificial* adj2 respirat*) or (artificial* adj2 ventilat*)) adj15 (length or prolonged or duration or time?)) or (ventilat* adj5 (day or days or hours))).mp. | 26016 |
| 43 | or/9-42 | 2577005 |
| 44 | (prognostic index or prognostic indices or prognostic rule* or prognostic abilit* or prognostic sign* or prognostic data or (laboratory value* or clinical value* or predictive abilit* or predictive values) or (indicator* and (predict* or prognos*)) or (predict* adj8 prognos*) or (laboratory value* or clinical value* or predictive abilit* or predictive values) or ((scor* or algorithm* or model* or variable* or machine learning) adj3 (predict* or prognos*)) or ((rule* or finding* or criteria or validat* or observ* or paramet*) adj3 (predict* or prognos*)) or (model* adj9 variable*) or (risk? adj5 (predict* or prognos*)) or ((mortalit* or survival) adj2 (predict* or prognos* or overpredict* or underpredict*)) or ((mortalit* or survival or outcome*) adj9 (indicator* or determinant*)) or ((multivariab* or multivariat* or multi-variab* or multi-variat* or univariab* or univariat* or logistic regression model* or logistic regression analys*) adj5 (prognos* or predict* or outcome* or endpoint* or end-point*)) or ((predict* or prognos* or indicator*) adj3 (recovery or functional)) or (("area* under the curve" or "area under curve" or "area under receive" or "area under the receiver" or "receiver operat* characteristic") and (prognos* or predict*)) or ((validation or stratification or discrimination or calibration or indices) adj15 (prognos* or predict*)) or ((predict* or prognos*) adj1 outcome*) or (prognostication* or clinical indicator* or clinical variable* or clinical characteristic* or clinical factor*) or ((factor* or characteristic* or feature*) adj3 (prognos* or predict*))).mp. | 1098308 |
| 45 | 8 and 43 and 44 | 5953 |
| 46 | limit 45 to english language | 5641 |
| 47 | exp animals/ not humans.sh. | 4963784 |
| 48 | 46 not 47 | 5538 |
| 49 | limit 48 to (address or bibliography or case reports or clinical conference or comment or congress or editorial or letter or news or newspaper article) | 137 |
| 50 | 48 not 49 | 5401 |
| 51 | limit 50 to ("review" or "systematic review") | 480 |
| 52 | 50 not 51 | 4921 |
| 53 | 52 not (address or bibliograph* or case report* or conference* or comment* or congress* or editorial* or letter* or news or newspaper*).mp. | 4836 |
| 54 | 53 not (Anecdote* or Biograph* or Book or Book Chapter* or Book Review* or, Brief Item* or Case Study or Directories or Dissertation or Thesis or Theses or Exam Questions or Pamphlet or Proceedings).af. | 4805 |

###### **SCOPUS = 4,355**

( ( ( ( TITLE-ABS-KEY ( ( brain* OR cerebr* OR cerebel* OR cortex OR crani* OR "Diffuse Axonal" OR head OR hemisphere* OR "Frontal Lobe*" OR "Temporal Lobe*" OR "Occipital Lobe*" OR "Parietal Lobe*" OR intracranial* ) W/0 ( injur* OR trauma* ) ) OR TITLE-ABS-KEY ( ( posttraumatic OR traumatic ) W/0 ( brain* OR encephalopath* OR intracrani* OR cerebel* OR cerebr* OR craniocerebr* ) ) OR TITLE-ABS-KEY ( ( traumatic OR posttraumatic ) W/0 ( brainstem OR bulbar OR medullary OR midbrain OR pontine OR subarachnoid OR subdural ) W/0 ( h?em?or*ag* OR h?ematoma* ) ) OR TITLE-ABS-KEY ( ( concuss* OR postconcuss* OR pneumocephalus OR "Crush* Skull*" OR "brain commotion" OR "Commotio Cerebri" OR "Contusio Cerebri" OR "posttraumatic coma" OR "post-traumatic coma" OR "depressed skull fracture" ) ) OR TITLE-ABS-KEY ( ( brain OR cerebr* OR cerebel* OR "Diffuse Axonal" OR intracrani* ) W/0 ( damage* OR wound* OR lacerat* OR contusion ) ) ) ) OR ( ( TITLE-ABS-KEY ( ( penetrat* W/2 skull* W/2 ( trauma* OR injur* OR fracture* ) ) ) OR TITLE-ABS-KEY ( ( penetrat* W/2 head* W/2 ( wound* OR fracture* ) ) ) OR TITLE-ABS-KEY ( ( ( severe OR moderate OR mild ) W/0 gcs ) ) OR TITLE-ABS-KEY ( ( "GCS score" W/1 ( "3" OR "4" OR "5" OR "6" OR "7" OR "8" OR "9" OR "10" OR "11" OR "12" ) ) ) OR TITLE-ABS-KEY ( ( ( "glasgow coma scale" OR "glasgow outcome scale" OR "glasgow scale" OR "Rancho Los Amigos" ) W/0 ( "3" OR "4" OR "5" OR "6" OR "7" OR "8" OR "9" OR "10" OR "11" OR "12" ) ) ) ) ) OR ( TITLE-ABS-KEY ( ( "traumatic lesion" W/2 ( brain OR cerebr* OR cerebel* OR cortex OR crani* OR "Diffuse Axonal" OR hemisphere* OR "Frontal Lobe*" OR "Temporal Lobe*" OR "Occipital Lobe*" OR "Parietal Lobe*" OR intracranial* OR encephalopath* OR bulbar OR medullary OR midbrain OR pontine OR subarachnoid OR subdural ) ) ) ) ) ) AND ( ( TITLE-ABS-KEY ( "physiological observation?" OR "clinical observation?" OR "cardiac rate?" OR "heart rate?" OR heartbeat? OR "pulse rate?" OR "vital sign?" OR "blood pressure" OR "diastolic pressure" OR "pulse pressure" OR "systolic pressure" ) ) OR ( TITLE-ABS-KEY ( "oxygen saturation*" OR "oxygen-h?emoglobin saturation" OR oximetries OR oximetry OR "pulse oximet*" OR anox?emia* OR anoxia OR hypox?emia OR hypoxia OR "oxygen deficienc*" OR "shock index*" OR "shock indices" ) ) OR ( TITLE-ABS-KEY ( "body temperature*" OR "organ temperature*" OR "heat loss*" OR thermoregulation* OR "skin temperature*" OR hypothermia* ) ) OR ( TITLE-ABS-KEY ( ( "pupil* size" OR "pupil* diameter*" OR "pupil* reflex" OR "pupil* light reflex" OR pupillometry OR "pupil* abnormalit*" OR "pupil* examination*" OR "pupil* measurement*" OR "pupil* response" OR "pupil* dilation" OR "pupil* constriction" OR "dilated pupils" ) ) OR ( ( pupil* W/1 ( react* OR unreactiv* ) ) ) ) OR ( TITLE-ABS-KEY ( "Glasgow coma scale*" OR "Glasgow coma score*" OR "GCS score" OR avpu OR "Alert and Verbal and Pain and Unresponsive" ) ) OR ( TITLE-ABS-KEY ( "blood alcohol" W/0 ( concentration* OR content OR level? OR reading? ) ) ) OR ( TITLE-ABS-KEY ( "body height" OR "body weight" OR obesity OR overweight OR underweight OR "body mass index" ) ) OR ( TITLE-ABS-KEY ( "coagulation factor*" OR fibrinogen* OR "coagulation paramet*" OR "fibrinolytic paramet*" OR "blood coagulation" OR "coagulation assay*" ) ) OR ( TITLE-ABS-KEY ( "Blood Platelet*" OR h?emoglobin* ) ) OR ( TITLE-ABS-KEY ( ( "international normali?ed ratio*" OR "INR level*" OR "INR value*" ) ) OR ( ( inr W/2 ( admission OR reversal ) ) ) ) OR ( TITLE-ABS-KEY ( ( ( "trauma severity" OR "injury severity" OR "Abbreviated injury" OR "Revised trauma" OR "Trauma injury severity" ) W/0 ( score* OR scale* OR index* OR indices OR measure* ) ) ) OR ( ( ( iss OR ais OR rts OR triss ) W/0 ( scor* OR scale* OR index* OR indices OR measure* ) ) ) OR TITLE-ABS-KEY ( apache OR "acute physiology and chronic health evaluation" OR "acute physiolog* assessment and chronic health evaluation" ) ) OR ( TITLE-ABS-KEY ( ( injur* OR trauma* ) W/2 ( characteristic* OR nature OR mechanism* ) ) OR TITLE-ABS-KEY ( associated W/0 ( injur* OR trauma* ) ) OR TITLE-ABS-KEY ( ( concurrent OR simultaneous* OR accompanying ) W/2 ( injur* OR trauma* ) ) ) OR ( TITLE-ABS-KEY ( ( "post-injury" OR postinjury OR "post-trauma*" OR posttrauma* ) W/2 ( complication* OR infection* ) ) ) OR ( TITLE-AB

S-KEY ( ( complication* OR infection ) W/2 ( after OR following ) W/2 ( injur* OR trauma* ) ) ) OR ( TITLE-ABS-KEY ( coagulopath* ) ) OR ( TITLE-ABS-KEY ( time W/1 ( transport OR scene ) ) OR TITLE-ABS-KEY ( "travel time*" OR "definitive care" OR "time to emergency" OR "time to hospital" OR "time to transport*" ) ) OR ( TITLE-ABS-KEY ( ( delay* OR optimal OR optimum ) W/2 ( time* OR timing ) ) OR TITLE-ABS-KEY ( ( time* OR timing ) W/2 ( intervention? OR treatment* ) ) ) OR ( TITLE-ABS-KEY ( ( "intensive care" OR "critical care" OR icu OR itu OR hdu ) W/4 ( day OR days ) ) OR TITLE-ABS-KEY ( ( icu OR itu OR hdu ) W/0 ( day OR days OR stay ) ) ) OR ( TITLE-ABS-KEY ( length W/1 ( hospital OR stay ) ) OR TITLE-ABS-KEY ( prolonged W/0 ( hospital* OR icu OR itu OR hdu ) ) ) OR ( TITLE-ABS-KEY ( ( primary OR secondary ) W/0 ( transport* OR transfer* ) ) OR TITLE-ABS-KEY ( patient? W/1 ( transport* OR transfer* ) ) OR TITLE-ABS-KEY ( interfacility OR interhospital OR "Inter-facility" OR "inter-hospital" ) ) OR ( TITLE-ABS-KEY ( ( ( "post-injury" OR postinjury OR "post-trauma*" OR posttrauma* ) W/2 amnes* ) ) OR ( ( amnes* W/2 ( after OR following ) W/2 ( injur* OR trauma* ) ) ) ) OR ( TITLE-ABS-KEY ( ventilat* W/4 ( day OR days OR hours ) ) ) ) AND ( TITLE-ABS-KEY ( "prognostic index" OR "prognostic indices" OR "prognostic rule*" OR "prognostic abilit*" OR "prognostic sign*" OR "prognostic data" OR "laboratory value*" OR "clinical value*" OR "predictive abilit*" OR "predictive values" ) OR TITLE-ABS-KEY ( indicator* AND ( predict* OR prognos* ) ) OR TITLE-ABS-KEY ( predict* W/6 prognos* ) OR TITLE-ABS-KEY ( ( scor* OR algorithm* OR model* OR variable* OR "machine learning" ) W/1 ( predict* OR prognos* ) ) OR TITLE-ABS-KEY ( ( rule* OR finding* OR criteria OR validat* OR observ* OR paramet* ) W/2 ( predict* OR prognos* ) ) OR TITLE-ABS-KEY ( model* W/7 variable* ) OR TITLE-ABS-KEY ( risk? W/3 ( predict* OR prognos* ) ) OR TITLE-ABS-KEY ( ( mortalit* OR survival ) W/1 ( predict* OR prognos* OR overpredict* OR underpredict* ) ) OR TITLE-ABS-KEY ( ( mortalit* OR survival OR outcome* ) W/8 ( indicator* OR determinant* ) ) OR TITLE-ABS-KEY ( ( multivariab* OR multivariat* OR "multi-variab*" OR "multi-variat*" OR univariab* OR univariat* OR "logistic regression model*" OR "logistic regression analys*" ) W/3 ( prognos* OR predict* OR outcome* OR endpoint* OR "end-point*" ) ) OR TITLE-ABS-KEY ( ( predict* OR prognos* OR indicator* ) W/2 ( recovery OR functional ) ) OR TITLE-ABS-KEY ( ( "area* under the curve" OR "area under curve" OR "area under receiver" OR "area under the receiver" OR "receiver operat* characteristic" ) AND ( prognos* OR predict* ) ) OR TITLE-ABS-KEY ( ( validation OR stratification OR discrimination OR calibration OR indices ) W/12 ( prognos* OR predict* ) ) OR TITLE-ABS-KEY ( ( predict* OR prognos* ) W/1 outcome* ) OR TITLE-ABS-KEY ( prognostication* OR "clinical indicator*" OR "clinical variable*" OR "clinical characteristic*" OR "clinical factor*" ) OR TITLE-ABS-KEY ( ( factor* OR characteristic* OR feature* ) W/2 ( prognos* OR predict* ) ) ) AND ( EXCLUDE ( SRCTYPE , "k" ) OR EXCLUDE ( SRCTYPE , "Undefined" ) ) AND ( EXCLUDE ( DOCTYPE , "re" ) OR EXCLUDE ( DOCTYPE , "cp" ) OR EXCLUDE ( DOCTYPE , "ch" ) OR EXCLUDE ( DOCTYPE , "le" ) OR EXCLUDE ( DOCTYPE , "ed" ) OR EXCLUDE ( DOCTYPE , "no" ) OR EXCLUDE ( DOCTYPE , "sh" ) OR EXCLUDE ( DOCTYPE , "cr" ) OR EXCLUDE ( DOCTYPE , "er" ) OR EXCLUDE ( DOCTYPE , "Undefined" ) ) AND ( EXCLUDE ( SUBJAREA , "ENGI" ) OR EXCLUDE ( SUBJAREA , "MULT" ) OR EXCLUDE ( SUBJAREA , "ARTS" ) OR EXCLUDE ( SUBJAREA , "AGRI" ) OR EXCLUDE ( SUBJAREA , "IMMU" ) OR EXCLUDE ( SUBJAREA , "DENT" ) OR EXCLUDE ( SUBJAREA , "COMP" ) OR EXCLUDE ( SUBJAREA , "CENG" ) OR EXCLUDE ( SUBJAREA , "ENVI" ) OR EXCLUDE ( SUBJAREA , "VETE" ) OR EXCLUDE ( SUBJAREA , "MATH" ) OR EXCLUDE ( SUBJAREA , "CHEM" ) OR EXCLUDE ( SUBJAREA , "MATE" ) OR EXCLUDE ( SUBJAREA , "PHYS" ) OR EXCLUDE ( SUBJAREA , "EART" ) OR EXCLUDE ( SUBJAREA , "DECI" ) ) AND ( LIMIT-TO ( LANGUAGE , "English" ) ) AND ( LIMIT-TO ( EXACTKEYWORD , "Humans" ) )

###### **SPORTDISCUS = 287**

| **#** | **Query** | **Results** |
| --- | --- | --- |
| S40 | S7 AND S37 AND S38  Limit English Language | 287 |
| S39 | S7 AND S37 AND S38 | 287 |
| S38 | (“prognostic index” or “prognostic indices” or “prognostic rule*” or “prognostic abilit*” or “prognostic sign*” or “prognostic data” or (“laboratory value*” or “clinical value*” or “predictive abilit*” or “predictive values”) or (indicator* and (predict* or prognos*)) or (predict* N7 prognos*) or ((scor* or algorithm* or model* or variable* or machine learning) N2 (predict* or prognos*)) or ((rule* or finding* or criteria or validat* or observ* or paramet*) N2 (predict* or prognos*)) or (model* N8 variable*) or (risk? N4 (predict* or prognos*)) or ((mortalit* or survival) N1 (predict* or prognos* or overpredict* or underpredict*)) or ((mortalit* or survival or outcome*) N8 (indicator* or determinant*)) or ((multivariab* or multivariat* or multi-variab* or multi-variat* or univariab* or univariat* or “logistic regression model*” or “logistic regression analys*”) N4 (prognos* or predict* or outcome* or endpoint* or “end-point*”)) or ((predict* or prognos* or indicator*) N2 (recovery or functional)) or (("area* under the curve" or "area under curve" or "area under receive" or "area under the receiver" or "receiver operat* characteristic") and (prognos* or predict*)) or ((validation or stratification or discrimination or calibration or indices) N14 (prognos* or predict*)) or ((predict* or prognos*) N1 outcome*) or (prognostication* or “clinical indicator*” or “clinical variable*” or “clinical characteristic*” or “clinical factor*”) or ((factor* or characteristic* or feature*) N2 (prognos* or predict*))) | 27,389 |
| S37 | S8 OR S9 OR S10 OR S11 OR S12 OR S13 OR S14 OR S15 OR S16 OR S17 OR S18 OR S19 OR S20 OR S21 OR S22 OR S23 OR S24 OR S25 OR S26 OR S27 OR S28 OR S29 OR S30 OR S31 OR S32 OR S33 OR S34 OR S35 OR S36 | 124,434 |
| S36 | ( (((mechanical* N1 ventilat*) or (mechanical* N1 respirat*) or (artificial* N1 respirat*) or (artificial* N1 ventilat*)) N14 (length or prolonged or duration or time?)) ) OR ( (ventilat* N4 (day or days or hours))) ) | 276 |
| S35 | ( (("post-injury" or postinjury or "post-trauma*" or posttrauma*) N2 amnes*) ) OR ( (amnes* N2 (after or following) N2 (injur* or trauma*)) ) | 185 |
| S34 | ( ((primary or secondary) N0 (transport* or transfer*)) ) OR ( (patient? N1 (transport* or transfer*)) ) OR ( (Interfacility or interhospital or "Inter-facility" or "inter-hospital") ) | 557 |
| S33 | ( (length N1 (hospital or stay)) ) OR ( (prolonged N0 (hospital* or ICU or ITU or HDU)) ) | 2,567 |
| S32 | ( (("intensive care" or "critical care" or ICU or ITU or HDU) N4 (day or days)) ) OR ( ((ICU or ITU or HDU) N0 (day or days or stay)) ) | 195 |
| S31 | ( ((delay* or optimal or optimum) N2 (time* or timing)) ) OR ( ((time* or timing) N2 (intervention? or treatment*)) ) | 3,958 |
| S30 | ( (time N1 (transport or scene)) ) OR ( ("travel time*" or "definitive care" or "time to emergency" or "time to hospital" or "time to transport*")) ) | 390 |
| S29 | Coagulopath* | 135 |
| S28 | ((complication* or infection) N2 (after or following) N2 (injur* or trauma*)) | 110 |
| S27 | (("post-injury" or postinjury or "post-trauma*" or posttrauma*) N2 (complication* or infection*)) | 56 |
| S26 | ( ((injur* or trauma*) N2 (characteristic* or nature or mechanism*)) ) OR ( (associated N0 (injur* or trauma*)) ) OR ( ((concurrent or simultaneous* or accompanying) N2 (injur* or trauma*)) ) | 5,093 |
| S25 | ( (("trauma severity" or "injury severity" or "Abbreviated injury" or "Revised trauma" or "Trauma injury severity") N0 (score* or scale* or index* or indices or measure*)) ) OR ( ((ISS or AIS or RTS or TRISS) N0 (scor* or scale* or index* or indices or measure*)) ) OR ( (APACHE or "acute physiology and chronic health evaluation" or "acute physiolog* assessment and chronic health evaluation")) ) | 1,429 |
| S24 | (MH "Apache") OR (MH "Trauma Severity Indices") | 33 |
| S23 | ( ("international normali?ed ratio*" or "INR level*" or "INR value*") ) OR ( (INR N2 (admission or reversal)) ) | 104 |
| S22 | ("Blood Platelet*" or h?emoglobin*) | 1,746 |
| S21 | ("coagulation factor*" or fibrinogen* or "coagulation paramet*" or "fibrinolytic paramet*" or "blood coagulation" or "coagulation assay*") | 1,017 |
| S20 | ("body height" or "body weight" or obesity or overweight or underweight or "body mass index") | 64,801 |
| S19 | (MH "Body Mass Index") OR (MH "Body Height") OR (MH "Body Weight") | 1 |
| S18 | ("blood alcohol" N0 (concentration* or content or level? or reading?)) | 373 |
| S17 | ("Glasgow coma scale*" or "Glasgow coma score*" or "GCS score" or AVPU or "Alert and Verbal and Pain and Unresponsive") | 616 |
| S16 | (MH "Glasgow Coma Scale") | 699 |
| S15 | ( ("pupil* size" or "pupil* diameter*" or "pupil* reflex" or "pupil* light reflex" or pupillometry or "pupil* abnormalit*" or "pupil* examination*" or "pupil* measurement*" or "pupil* response" or "pupil* dilation" or "pupil* constriction" or "dilated pupils") ) OR ( (pupil* N1 (react* or unreactiv*)) ) | 268 |
| S14 | (MH "Reflex, Pupillary") | 29 |
| S13 | ("body temperature*" or "organ temperature*" or "heat loss*" or thermoregulation* or "skin temperature*" or hypothermia*) | 7,357 |
| S12 | (MH "Body Temperature Regulation") OR (MH "Body Temperature") OR (MH "Core Body Temperature") OR (MH "Skin Temperature") OR (MH "Hypothermia") OR (MH "Hypothermia, Induced") | 5 |
| S11 | ("oxygen saturation*" or "oxygen-h?emoglobin saturation" or oximetries or oximetry or "pulse oximet*" or anox?emia*or anoxia or hypox?emia or hypoxia or "oxygen deficienc*" or "shock index*" or "shock indices") | 5,059 |
| S10 | (MH "Oximetry") OR (MH "Pulse Oximetry") | 210 |
| S9 | ("physiological observation?" or "clinical observation?" or "cardiac rate?" or "heart rate?" or heartbeat? or "pulse rate?" or "vital sign?" or "blood pressure" or "diastolic pressure" or "pulse pressure" or "systolic pressure") | 41,026 |
| S8 | (MH "Vital Signs") OR (MH "Blood Pressure Determination") OR (MH "Body Temperature Determination") OR (MH "Pulse") | 148 |
| S7 | S1 OR S2 OR S3 OR S4 OR S5 OR S6 | 15,275 |
| S6 | ("traumatic lesion*" N2 (Brain* or Cerebr* or Cerebel* or Cortex or Crani* or "Diffuse Axonal" or Head or Hemisphere* or "Frontal Lobe*" or "Temporal Lobe*" or "Occipital Lobe*" or "Parietal Lobe*" or intracranial* or encephalopath* or bulbar or medullary or midbrain or pontine or subarachnoid or subdural)) | 1 |
| S5 | ( ((severe or moderate or mild) N0 GCS) ) OR ( ("GCS score" N2 ("3" or "4" or "5" or "6" or "7" or "8" or "9" or "10" or "11" or "12")) ) OR ( (("glasgow coma scale*" or "glasgow outcome scale" or "glasgow scale*" or "Rancho Los Amigos") N0 ("3" or "4" or "5" or "6" or "7" or "8" or "9" or "10" or "11" or "12")) ) | 21 |
| S4 | ( (penetrat* N2 skull* N2 (trauma* or injur* or fracture*)) ) OR ( (penetrat* N2 head* N2 (wound* or fracture*)) ) | 4 |
| S3 | ( (Concuss* or Postconcuss* or Pneumocephalus or "Crush* Skull*" or "brain commotion?" or "Commotio Cerebri" or "Contusio Cerebri" or "posttraumatic coma*" or "post-traumatic coma*" or "depressed skull fracture*") ) OR ( ((Brain* or Cerebr* or Cerebel* or "Diffuse Axonal" or intracrani*) N0 (damage* or wound* or lacerat* or contusion?)) ) | 7,116 |
| S2 | ( ((Brain* or Cerebr* or Cerebel* or Cortex or Crani* or "Diffuse Axonal" or Head or Hemisphere* or "Frontal Lobe*" or "Temporal Lobe*" or "Occipital Lobe*" or "Parietal Lobe*" or intracranial*) N1 (Injur* or Trauma*)) ) OR ( ((Posttraumatic or Traumatic) N0 (brain* or encephalopath* or intracrani* or cerebel* or cerebr* or craniocerebr*)) ) OR ( ((traumatic or posttraumatic) N0 (brainstem or bulbar or medullary or midbrain or pontine or subarachnoid or subdural) N0 (h?em?or*ag* or h?ematoma*)) ) | 10,884 |
| S1 | (MH "Brain Injuries") OR (MH "Brain Concussion") OR (MH "Postconcussion Syndrome") OR (MH "Brain Contusions") OR (MH "Chronic Traumatic Encephalopathy") OR (MH "Pneumocephalus") OR (MH "Left Hemisphere Injuries") OR (MH "Right Hemisphere Injuries") | 14 |

######

###### **WEB OF SCIENCE CLINICAL INDICATORS SEARCH = 4081**

Query #1

((brain* OR cerebr* OR cerebel* OR cortex OR crani* OR "Diffuse Axonal" OR head OR hemisphere* OR "Frontal Lobe*" OR "Temporal Lobe*" OR "Occipital Lobe*" OR "Parietal Lobe*" OR intracranial* ) NEAR/0 ( injur* OR trauma*)) (Topic) or ((posttraumatic OR traumatic ) NEAR/0 ( brain* OR encephalopath* OR intracrani* OR cerebel* OR cerebr* OR craniocerebr*)) (Topic)

Query #2

((traumatic OR posttraumatic ) NEAR/0 ( brainstem OR bulbar OR medullary OR midbrain OR pontine OR subarachnoid OR subdural ) NEAR/0 ( h?em?orrag* OR h?ematoma*)) (Topic) or ((concuss* OR postconcuss* OR pneumocephalus OR "Crush* Skull*" OR "brain commotion" OR "Commotio Cerebri" OR "Contusio Cerebri" OR "posttraumatic coma" OR "post-traumatic coma" OR "depressed skull fracture")) (Topic) or ((brain OR cerebr* OR cerebel* OR "Diffuse Axonal" OR intracrani* ) NEAR/0 ( damage* OR wound* OR lacerat* OR contusion)) (Topic) or (penetrat* NEAR/2 skull* NEAR/2 ( trauma* OR injur* OR fracture*)) (Topic) or (penetrat* NEAR/2 head* NEAR/2 ( wound* OR fracture*)) (Topic)

Query #3

((severe OR moderate OR mild) NEAR/0 gcs) (Topic) or ("GCS score" NEAR/0 ("3" OR "4" OR "5" OR "6" OR "7" OR "8" OR "9" OR "10" OR "11" OR "12")) (Topic) or (("glasgow coma scale" OR "glasgow outcome scale" OR "glasgow scale" OR "Rancho Los Amigos" ) NEAR/0 ( "3" OR "4" OR "5" OR "6" OR "7" OR "8" OR "9" OR "10" OR "11" OR "12")) (Topic) or ("traumatic lesion" NEAR/2 (brain OR cerebr* OR cerebel* OR cortex OR crani* OR "Diffuse Axonal" OR hemisphere* OR "Frontal Lobe*" OR "Temporal Lobe*" OR "Occipital Lobe*" OR "Parietal Lobe*" OR intracranial* OR encephalopath* OR bulbar OR medullary OR midbrain OR pontine OR subarachnoid OR subdural)) (Topic)

Query #4

#1 OR #2 OR #3

#5

((((TS=(( "physiological observation?" OR "clinical observation?" OR "cardiac rate?" OR "heart rate?" OR heartbeat? OR "pulse rate?" OR "vital sign?" OR "blood pressure" OR "diastolic pressure" OR "pulse pressure" OR "systolic pressure" ))) OR TS=(( "oxygen saturation*" OR "oxygen-h?emoglobin saturation" OR oximetries OR oximetry OR "pulse oximet*" OR anox?emia* OR anoxia OR hypox?emia OR hypoxia OR "oxygen deficienc*" OR "shock index*" OR "shock indices" ) )) OR TS=(( "body temperature*" OR "organ temperature*" OR "heat loss*" OR thermoregulation* OR "skin temperature*" OR hypothermia* ) )) OR TS=(( "pupil* size" OR "pupil* diameter*" OR "pupil* reflex" OR "pupil* light reflex" OR pupillometry OR "pupil* abnormalit*" OR "pupil* examination*" OR "pupil* measurement*" OR "pupil* response" OR "pupil* dilation" OR "pupil* constriction" OR "dilated pupils" ) )) OR TS=(( pupil* NEAR/1 ( react* OR unreactiv* ) ) )

#6

(((((((((TS=(( "Glasgow coma scale*" OR "Glasgow coma score*" OR "GCS score" OR avpu OR "Alert and Verbal and Pain and Unresponsive" ) )) OR TS=(( "blood alcohol" NEAR/0 ( concentration* OR content OR level? OR reading? ) ) )) OR TS=(( "body height" OR "body weight" OR obesity OR overweight OR underweight OR "body mass index" ) )) OR TS=(( "coagulation factor*" OR fibrinogen* OR "coagulation paramet*" OR "fibrinolytic paramet*" OR "blood coagulation" OR "coagulation assay*" ) )) OR TS=(( "Blood Platelet*" OR h?emoglobin* ) )) OR TS=(( "international normali?ed ratio*" OR "INR level*" OR "INR value*" ) )) OR TS=(( inr NEAR/2 ( admission OR reversal ) ))) OR TS=(( ( "trauma severity" OR "injury severity" OR "Abbreviated injury" OR "Revised trauma" OR "Trauma injury severity" ) NEAR/0 ( score* OR scale* OR index* OR indices OR measure* ) ))) OR TS=(( ( iss OR ais OR rts OR triss ) NEAR/0 ( scor* OR scale* OR index* OR indices OR measure* ) ))) OR TS=(( apache OR "acute physiology and chronic health evaluation" OR "acute physiolog* assessment and chronic health evaluation" ) )

#7

((((((((((((((TS=(( ( injur* OR trauma* ) NEAR/2 ( characteristic* OR nature OR mechanism* ) ))) OR TS=(( associated NEAR/0 ( injur* OR trauma* ) ) )) OR TS=(( ( concurrent OR simultaneous* OR accompanying ) NEAR/2 ( injur* OR trauma* ) ) )) OR TS=(( ( "post-injury" OR postinjury OR "post-trauma*" OR posttrauma* ) NEAR/2 ( complication* OR infection* ) ) )) OR TS=(( ( complication* OR infection ) NEAR/2 ( after OR following ) NEAR/2 ( injur* OR trauma* ) ) )) OR TS=(coagulopath* )) OR TS=(( time NEAR/1 ( transport OR scene ) ) )) OR TS=(( "travel time*" OR "definitive care" OR "time to emergency" OR "time to hospital" OR "time to transport*" ))) OR TS=(( ( delay* OR optimal OR optimum ) NEAR/2 ( time* OR timing ) ) )) OR TS=(( ( time* OR timing ) NEAR/2 ( intervention? OR treatment* ) ) )) OR TS=(( ( "intensive care" OR "critical care" OR icu OR itu OR hdu ) NEAR/4 ( day OR days ) ) )) OR TS=(( ( icu OR itu OR hdu ) NEAR/0 ( day OR days OR stay ) ) )) OR TS=(( length NEAR/1 ( hospital OR stay ) ) )) OR TS=(( prolonged NEAR/0 ( hospital* OR icu OR itu OR hdu ) ) ))

#8

((((((TS=(( ( primary OR secondary ) NEAR/0 ( transport* OR transfer* ) ) )) OR TS=(( patient? NEAR/1 ( transport* OR transfer* ) ) )) OR TS=(( interfacility OR interhospital OR "Inter-facility" OR "inter-hospital" ))) OR TS=(( ( "post-injury" OR postinjury OR "post-trauma*" OR posttrauma* ) NEAR/2 amnes* ))) OR TS=(( amnes* NEAR/2 ( after OR following ) NEAR/2 ( injur* OR trauma* ) ) )) OR TS=(( ventilat* NEAR/4 ( day OR days OR hours ) ))) OR TS=(("artificial respirat*" NEAR/4 ( day OR days OR hours ) ))

#9

#8 OR #7 OR #6 OR #5

#10

(((((((((((((((TS=(( "prognostic index" OR "prognostic indices" OR "prognostic rule*" OR "prognostic abilit*" OR "prognostic sign*" OR "prognostic data" OR "laboratory value*" OR "clinical value*" OR "predictive abilit*" OR "predictive values" ) )) OR TS=(( indicator* AND ( predict* OR prognos* ) ))) OR TS=(( predict* NEAR/6 prognos* ) )) OR TS=(( ( scor* OR algorithm* OR model* OR variable* OR "machine learning" ) NEAR/1 ( predict* OR prognos* ) ) )) OR TS=(( ( rule* OR finding* OR criteria OR validat* OR observ* OR paramet* ) NEAR/2 ( predict* OR prognos* ) ) )) OR TS=(( model* W/7 variable* ) )) OR TS=(( risk? NEAR/3 ( predict* OR prognos* ) ) )) OR TS=(( ( mortalit* OR survival ) NEAR/1 ( predict* OR prognos* OR overpredict* OR underpredict* ) ) )) OR TS=(( ( mortalit* OR survival OR outcome* ) NEAR/8 ( indicator* OR determinant* ) ))) OR TS=(( ( multivariab* OR multivariat* OR "multi-variab*" OR "multi-variat*" OR univariab* OR univariat* OR "logistic regression model*" OR "logistic regression analys*" ) NEAR/3 ( prognos* OR predict* OR outcome* OR endpoint* OR "end-point*" ) ) )) OR TS=(( ( predict* OR prognos* OR indicator* ) NEAR/2 ( recovery OR functional ) ))) OR TS=(( ( "area* under the curve" OR "area under curve" OR "area under receiver" OR "area under the receiver" OR "receiver operat* characteristic" ) AND ( prognos* OR predict* ) ) )) OR TS=(( ( validation OR stratification OR discrimination OR calibration OR indices ) NEAR/12 ( prognos* OR predict* ) ) )) OR TS=(( ( predict* OR prognos* ) NEAR/1 outcome* ) )) OR TS=(( prognostication* OR "clinical indicator*" OR "clinical variable*" OR "clinical characteristic*" OR "clinical factor*" ) )) OR TS=(( ( factor* OR characteristic* OR feature* ) NEAR/2 ( prognos* OR predict* ) ) )

#11

#10 AND #9 AND #4
